# Supplementary material for: Single molecule turnover of fluorescent ATP by myosin and actomyosin unveil elusive enzymatic mechanisms
Source: Commun Biol. 2021 Jan 13;4:64. doi: 10.1038/s42003-020-01574-0 (PMC7806905; doi:10.1038/s42003-020-01574-0)
Supplement: Supplementary file 2 — Supplementary Information [file 42003_2020_1574_MOESM2_ESM.docx]

Single molecule turnover of fluorescent ATP by myosin and actomyosin unveil elusive enzymatic mechanisms

by

Marko Ušaj, Luisa Moretto, Venukumar Vemula, Aseem Salhotra and Alf Månsson

Supplementary Information

Contents

[List of Figures 3](#_Toc55290006)

[List of Tables 3](#_Toc55290007)

[1. Supplementary Materials and Methods 4](#_Toc55290008)

[1.1 Materials 4](#_Toc55290009)

[1.2 Buffers and solutions 4](#_Toc55290010)

[1.3 TIRF Microscopy 5](#_Toc55290011)

[1.4 Surface preparations 7](#_Toc55290012)

[1.5 In vitro motility assays 10](#_Toc55290013)

[1.6 Alexa647-ATP 11](#_Toc55290014)

[1.7 Photobleaching of Alexa647 11](#_Toc55290015)

[1.8 Myosin deposition for single molecule assays 12](#_Toc55290016)

[1.8.1 Simple myosin deposition 12](#_Toc55290017)

[1.8.2 Optimized myosin deposition 13](#_Toc55290018)

[1.9 Summary of optimized approach for TIRF based ATPase assays. 14](#_Toc55290019)

[1.10 Details of data Analysis 14](#_Toc55290020)

[1.11 Equilibrium dialysis 15](#_Toc55290021)

[1.12 Modeling of ATP binding 16](#_Toc55290022)

[2. Supplementary Results and Discussion 17](#_Toc55290023)

[2.1 Effects of different laser power 17](#_Toc55290024)

[2.2 Optimizing the assay buffer for TIRF experiments 19](#_Toc55290025)

[2.3 Specific versus nonspecific binding of Alexa647–ATP and Alexa647 moiety to assay surface. 23](#_Toc55290026)

[2.4 S1 and HMM ATPase under optimized conditions 25](#_Toc55290027)

[2.5 Effects of Alexa-ADP 29](#_Toc55290028)

[2.6 Blocking the myosin active site by fluorescent Alexa-ATP in the presence of vanadate. 30](#_Toc55290029)

[2.7 Equilibrium dialysis experiments 32](#_Toc55290030)

[2.8 Effect of ionic strength on Alexa-ATP on-dwell time distributions 35](#_Toc55290031)

[2.9 Modeling of ATP binding 36](#_Toc55290032)

[3. Supplementary References 39](#_Toc55290034)

# List of Figures

[**Fig. S1. Schematic of set-up for TIRF and EPI fluorescence microscopy.** 7](#_Toc49778337)

[**Fig. S2. Cleaning of glass coverslip surfaces and selection of BSA.**. 9](#_Toc49778338)

[**Fig. S3. Dependence of the processes detected in cumulative frequency distribution of Alexa647-nucleotide dwell-time events on the laser power (in milliwatts, mW).**. 19](#_Toc49778339)

[**Fig. S4. Stabilizing Alexa647 dye photophysics.**. 20](#_Toc49778340)

[**Fig. S5. Stabilizing the Alexa647 dye signal.**. 22](#_Toc49778341)

[**Fig. S6. Specific vs unspecific binding of Alexa-ATP and Alexa647 moiety (using Alexa647-cadaverine).**. 24](#_Toc49778342)

[**Fig. S7**. **Reproducibility of optimized single molecule ATPase assay.**. 26](#_Toc49778343)

[**Fig. S8. Double-exponential vs Triple exponential fit to cumulative frequency distributions for Alexa-nucleotide on-time events.**. 26](#_Toc49778344)

[**Fig. S9.** **Reproducibility of optimized single molecule actomyosin ATPase assay.** 28](#_Toc49778345)

[**Fig. S10.** **Single molecule HMM basal ATPase: role of Alexa-ADP.**. 29](#_Toc49778346)

[**Fig. S11. Representative time traces of Alexa-ATP binding to fluorescent S1*aD·Vi complex hotspot consistent with unspecific Alexa-ATP binding to myosin outside the active site.** 31](#_Toc49778347)

[**Fig. S12. Supportive data for Fig. 5 (main text) to determine the origin of “unexplained” (0.2-0.5 s^-1^) exponential phase.** 32](#_Toc49778348)

[**Fig. S13. Calibration of the equilibrium dialysis experiments.** 33](#_Toc49778349)

[**Fig. S14: Equilibrium dialysis experiments.** 34](#_Toc49778350)

[**Fig. S15. Effect of assay buffer ionic strength on observed processes in TIRF based basal myosin ATPase assay.**. 35](#_Toc49778351)

[**Fig. S16. Surface availability on BSA and myosin II S1 for ATP binding as calculated using ATPint for different thresholds.** 38](#_Toc49778352)

# List of Tables

**Table S1: Summary of best mean parameter values in double, triple, or triple with weighting (1/time) exponential fits.** ………………………………………………………...27

**Table S2: Comparison of different tools to detect ATP binding sites on myosin………..**36

# 1. Supplementary Materials and Methods

## 1.1 Materials

Para-aminoblebbistatin (AmBleb) was from Optopharma. Rhodamine Phalloidin (cat. no. R415), Alexa Fluor647-Phalloidin (cat. no. A22287), Alexa Fluor647-ATP (cat. no. A22362) and Alexa Fluor647-cadaverine were obtained from Thermo Fisher Scientific. Trolox (cat. no. 238813), cyclooctatetraene (COT, cat. no. 138924), 4-Nitrobenzyl alcohol (NBA, cat. no. N12821), pyranose oxidase (POX, cat. no. P4234), bovine serum albumin (BSA, standard purity, cat. no. A2153), bovine serum albumin (BSA, high purity, cat. no. A0281), dithiothreitol (DTT), glucose oxidase (GOX, cat. no. G2133), catalase (cat. no. C100), creatine phosphate (PK, cat. no. P7936), adenosine triphosphate (ATP, cat. no. A2383), creatine phosphokinase (CPK, cat.no. C3755), MOPS, KCl, MgCl_2_, K_2_EGTA, HCl, KOH, Liquinox, Ethanol, Methanol, Glucose, DMSO, Sodium Orthovanadate were purchased from Sigma Aldrich (now Merck). Other biochemical reagents were of analytical grade and purchased from Sigma Aldrich.

## 1.2 Buffers and solutions

Stock solutions of chemicals were prepared according to user manuals or published literature. In order to minimize solvent concentrations in the final assay solution, COT and NBA were prepared together in DMSO as 200 mM stock, aliquoted and kept at -20 °C. Trolox was prepared on the day of the experiment in a low ionic strength solution (LISS, see below) at final concentration of ~2 mM. Due to poor solubility of Trolox in water, the powder was first dissolved in methanol as a 100 mM solution and subsequently diluted with LISS buffer^1^. pH was adjusted to 7.4 by KOH titration. The Trolox solution was filtered (0.2 µm) into a 10 cm Petri dish and exposed to UV-light (254 nm) to form Trolox-Quinone^1^. Our optimized exposure at 120,000 µJ/cm^2^ for 15 min using Stratalinker1800 (Stratagene) yielded ~20 % Trolox-Quinone in solution (molar ratio). Finally, a Trolox-Trolox/Trolox Quinone mixture in LISS (***TX/TQ-LISS***) was degassed for use in assay solutions (see below).

A low ionic strength solution (LISS; pH 7.4) contained 10 mM MOPS, 1 mM MgCl_2_ and 0.1 mM K_2_EGTA. A **wash buffer** was prepared to contain 50 mM KCl and 1 mM DTT in LISS. Standard assay solution^2,3^ (IVMA buffer) for gliding in vitro motility assays was prepared in LISS with addition of (final concentrations) 10 mM DTT, 45 mM KCl, 3 mg/ml glucose, 0.1 mg/ml glucose oxidase, 0.01 mg/ml catalase, 2.5 mM creatine phosphate, 0.2 mg/ml creatine phosphokinase and 1 mM MgATP. ***Optimized TIRF assay solution (TIRF buffer)*** was prepared in TX/TQ-LISS (see above) with 45 mM KCl, 10 mM DTT, 7.2 mg/ml glucose, 3 U/ml POX, 0.01 mg/ml catalase, 2.5 mM CP, 0.2 mg/ml CPK, 2 mM COT, 2 mM NBA, ~2 mM TX/TQ and 0.64% methylcellulose with or without Alexa647-ATP (up to 10 nM). Note, it is important first to add COT and NBA to TX/TQ-LISS and vortex to ensure proper solubility of these two components. Ionic strength of the assay buffer was 60 mM.

The traditionally used oxygen scavenger system based on glucose oxidase - GOC (glucose/glucose oxidase/catalase) was replaced by pyranose oxidase – POC (glucose/pyranose oxidase/catalase) with the aim to prevent acidification of the assay buffer under prolonged observation^4^. Catalase and pyranose oxidase were prepared in LISS as 100X solution followed by centrifugation (10,000×g, 1 min) and storage of the supernatant at 4 °C for up to four weeks.

Methylcellulose was prepared at 1.6 % (w/v) in TX/TQ-LISS, stirred overnight at 4 °C, degassed, aliquoted and stored at -20 °C.

AmBleb was prepared in DMSO, aliquoted and stored at -20 °C. The stock concentration was checked by absorbance measurements on spectrophotometer (UV1800, Shimadzu) using an extinction coefficient of 6860 M^-1^cm^-1^ (aqueous solution at pH = 7.3) according to the manufacturer´s instructions. DTT (1 M) was prepared freshly on the day of the experiments in LISS. Activated sodium orthovanadate (vanadate further in text) was prepared in water by repetitive steps of adjusting pH-boiling-cooling^5,6^. The activation was monitored by observing change of the solution color from yellow to colorless. The final pH of the 52.5 mM sodium orthovanadate stock (as determined spectrophotometrically using extinction coefficient ε = 3,550 at 260 nm) was stabilized at pH 8.0. The stock was then aliquoted and stored at -20 °C.

## 1.3 TIRF Microscopy

We used an objective type total internal reflection fluorescence (TIRF) microscope for all single molecule experiments. The TIRF microscope was custom-built around a Nikon epifluorescence inverted microscope Nikon Eclipse TE300 arranged on an optical table (CleanBench 63-574, TMC). A 60X oil immersion objective lens with high numerical aperture was used (CFI Apochromat TIRF 60XC Oil, NA 1.49, Oil; Nikon) and images were recorded using an EMCCD camera (Andor iXon Ultra 897 EMCCD) and NIS Elements software (Nikon, ver. 4.51, gain parameter = 100). Image depth at recording was 16-bit but was later converted to 8-bit to reduce computer time and space needed for processing, analyzing and storage. The exposure time was set to 50 ms, yielding the average time between frames ~52 ms, limiting the frame rate to 19.33 fps to allow continuous recording for sufficient time (900-1800 s). Pixel size of acquired images was 0.267×0.267 µm^2^. Red lasers (Melles Griot, 56RCS/S2799, OEM diode laser 45 mW, 642 nm or Melles Griot 05-LHP-925 30 mW, 632.8nm HeNe lasers) were used to excite Alexa647.

The optical set-up is based on published design criteria^7,8^. Speckle across the field of view was minimized, using a vibrating optical fiber, to provide as uniform illumination as possible. The laser beam was coupled into the optic fiber that was rolled around the vibrator unit. The latter was realized either as an electric toothbrush (Oral-B Pro60C) or, more effectively, as a “hanging” vortex mixer (Vortex Genie 2, Scientific Industries) with custom-adapted head, vibrating at maximal speed.

Our TIRF-microscope is depicted in Fig. S1. The laser beam was coupled into the multimodal optical fiber (MMF, M14L02 - Ø50 µm, 0.22 NA, SMA-SMA Fiber Patch Cable, Low OH, 2 Meters, ThorLabs), mounted on a fiber holder (H1, SM1SMA fiber adaptor, ThorLabs), with the help of a focusing lens (L1, AC254-030-A-ML, ThorLabs). The Optical fiber was guided around the fiber vibrator (see above) and mounted on a Fiber Port (PAF-SMA-5-A, ThorLabs). With the help of a collimating lens (L2, f = 250 mm, ThorLabs) the beam was collimated and finally, using the lens L3 (AC254-250-A-ML, ThorLabs), it was focused to the objective back focal plane. Using a manual mirror, M3 on a 1D stage we achieved TIRF illumination by positioning the beam on the outer edge of the back focal plane. The same stage allows removal of the mirror from the optical path to achieve epifluorescence illumination (100 Watt Mercury Lamp) for imaging of Rhodamine phalloidin labeled actin filaments. Careful alignments of the optical elements was aided by power measurements using the USB Power and Energy Meter device (PM100USB, ThorLabs) equipped by Silicon Power Head (400-1100 nm, 50 mW, ThorLabs), controlled by dedicated software provided by the manufacturer. Maximal power measured at the back focal plane position was ~2.7 mW (diode laser), 2.5 mW (for HeNe laser #1) or ~0.7 mW (for HeNe laser #2). We were able to reduce power stepwise to ~25 %, ~13% or ~4 % of maximal power by the use of Neutral Density filters. To achieve satisfactory signal to noise ratio (SNR), without noticeable increase in the bleaching rate, the experiments were performed at maximal power (HeNe laser #2) or at ~0.5-0.7 mW power (NeHe laser #1 and diode laser, respectively, with ND filter in use) unless stated otherwise. In our experimental set-up, the illuminated area was disc-like with approximately 40 µm diameter. Filter cubes were used to select a suitable wavelength range according to the emission spectra of the fluorophores (Cy5 for Alexa 647 and TRITC for Rhodamine; Epi-FL Filtersets, Nikon).


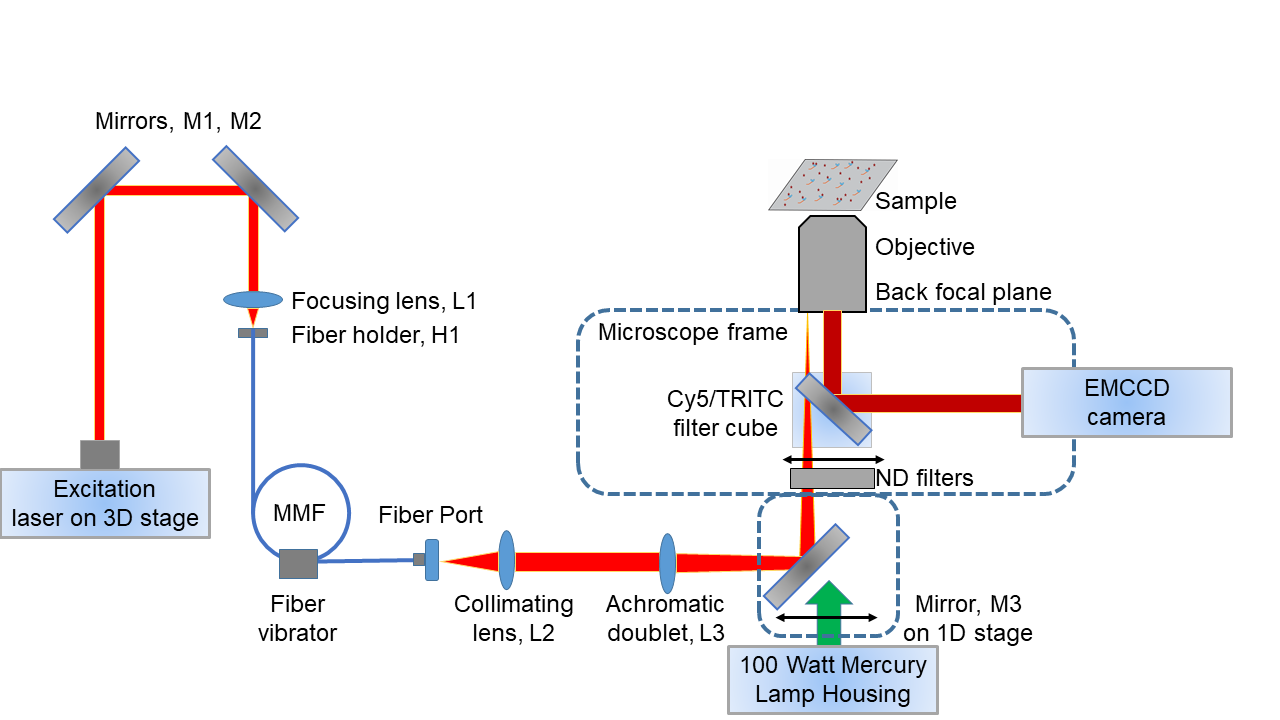


**Fig. S1. Schematic of set-up for TIRF and EPI fluorescence microscopy.**

## 1.4 Surface preparations

Glass coverslips used for single molecule assays need to be particularly clean. Out of the box coverslips usually contain numerous unidentified fluorescent objects (UFOs, Fig. S2a). However, it seems that the density of UFOs and their behavior varies in a random fashion from coverslip to coverslip and from batch to batch. We could not identify the sources of the UFOs but most commercial cover slips are flame-polished, a procedure that could potentially generate fluorescent nanoparticles with blinking behavior. The coverslips were cleaned in several steps. Coverslips were placed in water-filled containers, one by one and then subjected to: 20 min sonication in Liquinox (1%) and ethanol (95 %), 20 min incubation in Aqua regia and 20 min sonication in KOH (2.5 M)^9^. Between different steps, coverslips were rinsed in water (3x). Aqua regia was prepared by mixing two parts of nitric acid with one part HCl^9^. Subsequently, coverslips were further derivatized with trimethylchlorosilane (TMCS) as described earlier^10,11^. Notably, this includes incubation of coverslips in piranha solution (5 min, 80 °C), which per-se did not efficiently remove UFOs. *Caution! Piranha solution is a highly corrosive acidic solution, which can react violently with organic materials. Do not store in closed container, and use appropriate safety precautions. Similarly, aqua regia solutions are extremely corrosive and may result in explosion or skin burns if not handled with extreme caution. Before disposal, the solution should be cooled down and neutralized with sodium bicarbonate. Never store aqua regia in a closed container.* In later experiments an alternative procedure was used where the coverslips were cleaned by air plasma ashing using plasma cleaner Femto Standard (Diener electronic GmbH, Germany), at 100 W (40 kHz), 0.6-0.8 mbar pressure, for 3 minutes. The plasma ashing produced coverslips with comparable cleanness as the above described chemical approach but the method was not accessible in early experiments. To passivate surfaces, high purity BSA was centrifuged (220,000×g, 15 min) before use^12^. This process yielded clean functionalized glass coverslips with few remaining UFOs (Fig. S2b). These were the type of slides that were used in our single molecule assays of ATP turnover. Fluorescence trajectories from remaining UFOs and non-specific Alexa-ATP binding to the surfaces are depicted in Fig S2c and Fig. S2d, respectively. The dwell times were extracted (as described further in text below) and plotted as cumulative distributions that can be fitted by double exponential functions (Fig. S2e-g). Surface preparations as described above, minimized the presence of UFOs and Alexa-ATP nonspecific binding. Notably, none of the analyzed traces from UFOs in Fig. S2e-f from properly prepared surfaces would pass the criteria for “hotspots” (see further text below) of having more than 10 events per 15 min trace.


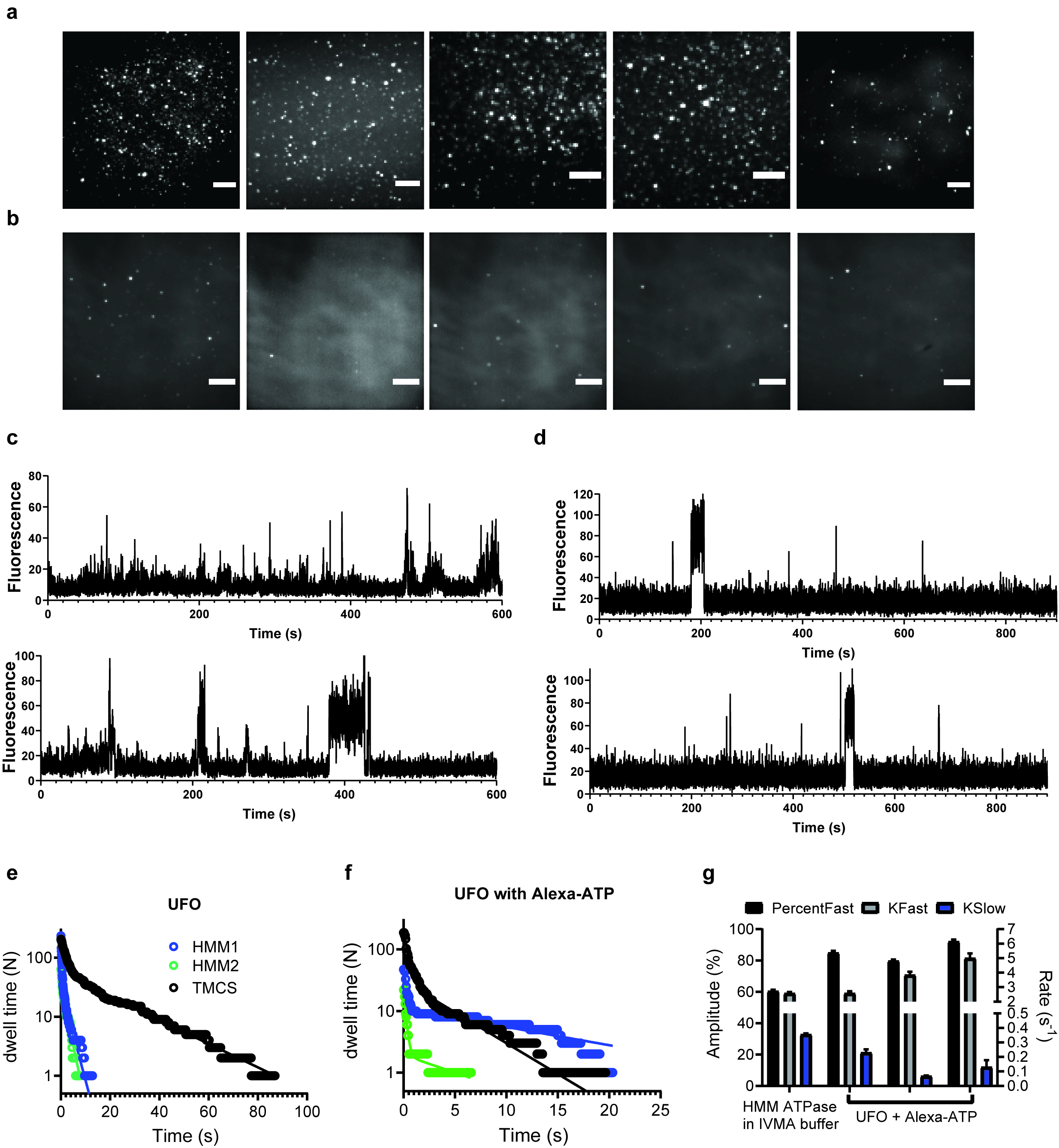


**Fig. S2. Cleaning of glass coverslip surfaces and selection of BSA.** Time projections and analyses of 15 min videos (50 ms exposure time/frame) from different glass slides recorded in TIRF assay buffer containing 5-10 nM Alexa647-ATP. Bars, 5 µm. **a** Out-of-the-box glass coverslips, cleaned with piranha solution, silanized and coated with standard grade BSA. **b** Glass coverslips cleaned using optimized multistep procedure described in Materials and Methods followed by cleaning with piranha solution, silanization and coating with selected, non-fluorescent BSA. **c** Two representative time traces of UFO fluorescence without Alexa-ATP. **d** Two representative time traces of UFO fluorescence on non-fluorescent high-purity BSA coated surfaces with Alexa-ATP present. **e** Cumulative dwell time distributions of UFOs (note, no Alexa-ATP present) from piranha cleaned surfaces derivatized with TMCS only (“TMCS”) or also subsequently incubated with 1 nM HMM and 1mg/ml standard BSA (“HMM1” ), or with 34.3 pM HMM and 1mg/ml non-fluorescent BSA (“HMM2”). Solid lines are fits to double exponential functions. The basis for the different appearance between TMCS and HMM-labelled plots could be effects of proteins on photophysics of UFOs or just random effects. Detailed analysis is outside the scope of the study. **f** Cumulative dwell time distributions of UFOs from non-fluorescent BSA-coated and optimally cleaned surfaces in the presence of Alexa-ATP (≤ 10 nM; three surfaces were examined) showing different behavior (seemingly random) from slide to slide. Solid lines are fittings by double exponential functions. **g** Rates and amplitudes obtained from fitting the data in f. Error estimates refer to 95 % confidence intervals derived in the regression analysis. Note how the values for UFOs are in similar ranges as seen for HMM ATPase studied in an IVMA buffer (as Fig. 2f, main paper). However, importantly, the bright spots to which data in Fig. S2f-g refer, would not pass the criteria for “hotspots” (at least 10 binding events per 15 min trace; see further text) to be included in studies of ATP turnover.

## 1.5 In vitro motility assays

The experiments were performed using flow-cells assembled with trimethylchlorosilane derivatized glass coverslip (#0 or #1, 24 × 60 mm) for the floor and untreated glass coverslip (#0, 18 x 18 mm^2^) for the ceiling of the cell, spaced (~100 μm) with double-sided tape (3M Scotch). The flow cell volume was 10-15 μl. For in vitro motility assays^3,13^, flow cells were incubated with heavy meromyosin (HMM; 120 μg/ml; 5 min) and then with BSA (1mg/ml; 2 min), both in Wash buffer. Subsequently, the flow cells were rinsed with Wash buffer, incubated with 2-10 nM rhodamine-phalloidin labeled actin filaments (prepared in Wash buffer) and rinsed again with Wash buffer, prior to addition of the assay solution (IVMA buffer). Image acquisition was performed using an inverted fluorescence microscope Zeiss (Axio Observer.D1, Zeiss, Germany, with a 63x objective, NA=1.4). For excitation, a Mercury short-arc lamp (OSRAM GmbH) was used together with suitable filter sets allowing observation of Rhodamine fluorescence. Image sequences were recorded using an electron multiplying charge-coupled device (EMCCD) camera (C9100-12PHX1, Hamamatsu Photonics) with pixel size (on sample) of 0.16x0.16 μm^2^ and 0.24x0.24 μm^2^ for 100x and 63x objective, respectively. The actin filament movements were recorded using a frame rate in the range 4-10 frames/second. Experiments were performed within the 23-25 °C temperature range but temperature was constant to within 1 °C during a given experiment (see further figure legends for details). Actin filament gliding velocities were calculated as described earlier^2,14^. The cut-off of the coefficient of variation (CV) (standard deviation of frame-to-frame velocity divided by average velocity in ten frames) for data inclusion in velocity analysis was set to 0.2.

## 1.6 Alexa647-ATP

We selected Alexa647–ATP as fluorescent ATP analogue because of extensive previous characterization in the lab^15^ showing similar sliding velocities in the IVMA and similar basal HMM-catalyzed MgATP turnover as standard non-fluorescent MgATP^15,16^. Furthermore, importantly, all four isomers of Alexa647-ATP were found to behave similarly with respect to basal myosin ATPase. Finally, the creatine-kinase/creatine phosphate ATP-regenerating system seems to be compatible with Alexa647-ADP^17^ which is useful in preventing accumulation of the latter product. This is in contrast to Cy3-labeled nucleotides which are not suitable substrates for the ATP-regenerating system^18^. On the other hand, and in contrast to Cy3–ATP, the fluorescence intensity of Alexa647-ATP is reduced upon binding to myosin^15^.

## 1.7 Photobleaching of Alexa647

The single molecule photobleaching rate of Alexa647 was determined using two assays: either actin filaments sparsely labeled with Alexa647-phalloidin (molar ratio Alexa647- phalloidin : actin subunits = 1:240) or Alexa647-ADP locked into the ATP-pocket by vanadate. Either 34.3 - 68.6 nM HMM or myosin subfragment 1 (S1) fragments were incubated in LISS supplemented with 1 mM DTT and 30 mM KCl with 4 µM AlexaATP in the presence of 20 µM vanadate for 3-4 h at room temperature (20 – 23 ^o^C). After that the sample was kept on ice, subsequently diluted to the desired concentration and used immeddiately.^16^. The latter approach is important as a supplement because it examines the dye photophysics in an environment similar to ATPase studies. Simple deposition of myosin-Alexa-ADP-vanadate complexes (employing HMM or S1) was used for these experiments. The time traces were extracted as described above with the help of time projection images. The total bleaching time was determined from events, which produced single step decrease in fluorescence intensity. We have also noted recurrence of a high fluorescence state suggesting dye transition back to the bright state from dark state. In such cases, we collected the times of the first step decrease in fluorescence intensity only. Fitting cumulative distributions of those events yielded single molecule photobleaching rate constants. Under our optimized conditions, the latter was usually an order of magnitude slower than that for the basal myosin ATPase (Fig. 2).

## 1.8 Myosin deposition for single molecule assays

Many functional assays benefit from the use of unmodified myosin molecules, i.e. without conjugation to a fluorescent dye or expression with a fluorescent protein. In this study we therefore used non-labeled myosin constructs. For this reason, it was essential to have high confidence that collected signals originate from myosin-associated events. We deployed several strategies to increase this confidence.

### 1.8.1 Simple myosin deposition

In the simple myosin deposition, we infused myosin motor fragments in pM concentrations to allow them to adsorb in random locations on the silanized surface. This procedure (Fig. 2a-b, i) is similar to that used in all (to the best of our knowledge) studies of single molecule myosin basal ATPase until now. The myosin motor fragments at such low concentration were prepared in wash solution further supplemented with 0.1 mg/ml of BSA to prevent myosin deterioration and protein loss through attachment to tube wall. Recorded videos were first subjected to simple background subtraction using the Fiji (ImageJ) function “Process/Subtract Background” with parameter “Rolling ball radius” set to five, successfully decreasing noise of free dye as well diminishing any illumination non-uniformities while preserving signal features. Time projection images of background-subtracted videos were created using the Fiji function “Image/Stack/Z-project/STD”. Brightness and contrast were adjusted by using the Fiji function “Image/Adjust/Brightness/Contrast”. These images were then used to manually select hotspots, i.e. regions of interest of 3 × 3 pixels with significant time (defined below) of high fluorescence intensity (ON-time). Using this approach, it is essential to start with clean surfaces. We used chambers with different densities of deposited myosin molecules. As shown in Fig. 2bi, the number of fluorescent spots in a field of view approximately scaled with the concentration of myosin in the solution infused into the assay chamber. To strengthen the probability that the selected spot is truly (active) myosin we only consider “hotspots” i.e. spots with at least 10 repetitive binding events (ON-times) during a 15 min recording; a criterion used previously^19^. We also employ previously suggested procedures to optimize the selection of specific binding events of individual Alexa647 molecules^19^. For example, we included only those signals for which both sudden one-step increase and sudden one-step decrease occurred while signals arising from more than one fluorophore beginning and ending in double- or even multiple-steps were excluded.

### 1.8.2 Optimized myosin deposition

As a novelty, we developed and used optimized myosin deposition via actin filaments (Fig. 2a-b, ii-iii). In this approach myosin motor fragments in pM concentrations (typically 85.75 pM) were preincubated with 40 nM actin filaments (G-actin concentration) labeled with Rhodamine-phalloidin in wash buffer supplemented with 0.1 mg/ml BSA for at least 10 min on ice to form actomyosin. Actomyosin was then infused into a flow cell to allow attachment to the silanized surface (5 min, RT). Subsequently, the flow cell was incubated with BSA (1 mg/ml, 2 min) and we obtained images of the actin filaments. To measure myosin basal ATPase activity, actin filaments were washed away by incubation with non-fluorescent MgATP (~ 100 µM) in wash buffer for 2 min following extensive washing with ATP-free wash buffer. Images of any remaining actin filaments were acquired. With this procedure, actin filaments were either completely removed or small actin filament fragments remained, presumably due to certain number of ATP insensitive heads (Fig. 2a-b, ii). These remaining filament fragments are potentially useful for alignment of images taken between different steps, e.g. to correct for possible drift due to media exchange, etc., (e.g. Fig S6d), or to even probe actomyosin ATPase under them. Finally, assay solution (IVMA or TIRF buffer) was injected into the chamber and a video was recorded. Time projected images of background-subtracted video recordings of nucleotide binding were merged with both images of the actin filaments using a Fiji function (“Image/Color/Merge channels”). Only hotspots co-localized with actin filaments before ATP wash and without actin filaments after ATP wash were considered for analysis of myosin basal ATPase. The optimized deposition of actin filaments also gives us excellent opportunity to reliably measure single molecule actin activated myosin ATPase for the first time. To this end, the actin filaments were not washed away after deposition. Rather, a gentle wash was performed with low [MgATP] of 100 nM to remove myosin motor fragments that were attached to the actin filament but not to the surface (Fig. 2a, ii; 2b, iii). The rinsing step enhanced single molecule detection by avoiding overlapping myosin heads in single ROIs of 3 × 3 pixels. Furthermore, the procedure also blocked the active sites of any non-functional myosin heads that showed irreversible ATP binding (without turnover activity). The latter could otherwise produce unrealistically long fluorescence ON events, limited only by fluorophore bleaching. Again, images of actin filaments were acquired before and after ATP wash. Only hotspots of Alexa647-ATP binding that co-localized with both actin images were considered for analysis of actin activated myosin ATPase.

## 1.9 Summary of optimized approach for TIRF based ATPase assays.

In order to remove the effect of UFOs, flow cell surfaces were extensively cleaned as described above and optimally selected and treated BSA was used for surface blocking. Generally, and unless otherwise stated, myosin motor fragments that were used for ATPase assays were deposited together with actin filaments to give highest confidence in defining the locations of the motors. Independent of mode of deposition, the analysis was limited to surface hotspots as defined above. Further, the assay was performed using ***optimized assay solution (TIRF buffer)*** containing: TX/TQ-LISS with 45 mM KCl, 10 mM DTT, 7.2 mg/ml glucose, 3 U/ml POX, 0.01 mg/ml catalase, 2.5 mM CP, 0.2 mg/ml CPK, 2 mM COT, 2 mM NBA, ~2 mM TX/TQ (TQ/TX = 0.2) and 0.64% methylcellulose with or without Alexa647-ATP (up to 10 nM). The basis for the optimized assay procedure is described in detail and further justified elsewhere in the paper.

## 1.10 Details of data Analysis

We followed recently laid out principles for analysis of dwell-time trajectories ^19-21^. For general image processing we have used Fiji^22-25^ with built-in functions as indicated in the text. Software routines were written in MATLAB (MathWorks, ver. 2017, 2018a, 2019b) for semi-automated analysis of Alexa-ATP dwell time recordings and to extract time traces of each individual fluorescence spot. The first step in the software routine is to allow the user to select fluorescence spots as well as nearby background ROIs on a time projection image, created as described above. An individual ROI is defined as a 3×3 pixel area. The integrated intensity of each ROI is calculated frame by frame for the entire sequence and stored as a time series. Beside batch generation of time traces from multiple ROIs we have also used manual (ROI by ROI) extraction using the Fiji function (Image/Stacks/Plot Z-axis Profile). These time series are finally analyzed manually to measure the dwell times of fluorescence spots, i.e., the time from a one-step increase of intensity above a pre-defined threshold until the single-step drop in intensity back below the intensity threshold. Only fluorescence events that start and end with a one step change in intensity were considered. In the optimized deposition of myosin, only those regions of interest were included that co-localized with an actin filament visualized by Rhodamine-phalloidin labeling (see above). The resulting dwell times were interpreted as the time spent by Alexa647-nucleotide (Alexa647-ATP and Alexa647-ADP) bound to an immobilized myosin molecule. Collected dwell times were plotted as cumulative distributions as described before^19^. The data were fitted using non-linear regression in GraphPad Prism (ver. 7 and 8). Reported rates and amplitudes represent mean ± 95% CI.

In analysis of actomyosin Alexa-ATPase data, a frameshift correction is necessary to correctly capture fast events without systematic errors with significant influence on the analysis. Thus, we systematically subtracted the time interval (52 ms) between two subsequent frames from all collected dwells for analysis of actomyosin. In this case, it produced slightly better fit (especially when analyzing single hotspots only, e.g. Fig. 4) and statistically significant increases of the faster rates associated with actomyosin ATP turnover (Fig. 2g-i, Fig S9). The implementation of this correction had negligible (statistically non-significant) effects on rate constants and amplitudes associated with basal Alexa-ATP turnover by myosin alone and the correction was not implemented for this condition.

## 1.11 Equilibrium dialysis

Equilibrium dialysis measurements were performed using Fast-Micro-Equilibrium Dialyzers^26^ with 25 μl chambers (cat.no. 7416-255D), and corresponding regenerated cellulose membranes of 10 or 25 kDa MW cut-off (cat.no. 7416-RC10K, 7416-RC25K), all from Harvard Apparatus (Harvard Apparatus, Boston, MA, USA). Prior to experiments, the HMM sample was pre-dialyzed into assay buffer (for ~3 h at 4 °C) using a Fast Spin dialyzer with 200 µl chamber (cat.no. 740508) with corresponding regenerated cellulose membranes of 10 kDa MW cut off membranes (cat. no. 7424-RC10K) both from Harvard Apparatus (Harvard Apparatus, Boston, MA, USA). The protein sample was used as is, or further diluted with assay buffer. For each experimental run sufficiently big aliquots of the protein sample was stored for re-evaluation of protein concentration. Assay buffer for equilibrium dialysis experiments was based on LISS (see above) with 5, 45 or 115 mM KCl and 1 mM DTT (ionic strengths of 20, 60, 130 mM, respectively, named as W20, W60 and W130) supplemented with desired concentration of Alexa-ATP and 10-fold molar excess of vanadate to inhibit any basal ATPase activity during the dialysis. Experiments were performed overnight at 4 °C on a moving platform. The concentration of free ligand (i.e. Alexa-ATP) was determined at the beginning of the experiment by measuring the absorbance at 650 nm (ε = 270,000 M^-1^ cm^-1^, T = 25 °C), and at the end of the dialysis from the ligand-only chamber. Bound ligand concentration was determined as before using a mass conservation equation^26^, modified to meet experimental specifics (see below) for labeled nucleotide: N_i_ = R·N_f_ + n[HMM], where N_i_ is the concentration of the nucleotide at the beginning of the dialysis and N_f_ is the final concentration at the end of the experiment, from the ligand only chamber. R is the ratio between [N]_i_/[N]_f, control_ in parallel control experiments without HMM. It was necessary to carefully determine the ratio R because we observed concentration imbalance and some ligand loss at equilibrium reached overnight when using fluorescent Alexa-ATP (unlike unlabeled ATP) in control experiments without HMM (Fig. S13). Thus for each condition we have always run in parallel two equilibrium dialysis experiments, one with HMM and one without HMM (control) to estimate the factor R which addresses any nucleotide loss (e.g. by adsorption to the dialysis membrane, etc.) and any imbalance at equilibrium reached overnight. We have deliberately avoided to run dialysis for longer time than that in order to prevent functional deterioration of the myosin molecules. The concentration imbalance at equilibrium in the absence of HMM was presumably due to electrostatic interactions between the dye and the membrane (as suggested by results at different ionic strengths, Fig. S13 B, C). From control runs we have also determined the apparent Alexa-ATP concentration which was effectively “felt” by HMM. This was the Alexa-ATP concertation in the initially nucleotide empty control chamber after overnight dialysis (denoted as [aT]_f, HMM-free_). All the calculated number of sites (n) were plotted against this (i.e. [aT]_f, HMM-free_) nucleotide concentration (and not against the initial concentration divided by 2, as would be valid for imbalance-free experiments). Again, with this approach we have effectively taken into account any dye loss and imbalance between the chambers. In the high concentration regime, [HMM] was held at 1.14 ± 0.1 µM (N=5, mean ± SD) and in the low concentration regime [HMM] was 304 ± 21 nM (N=5, mean ± SD), but constant during individual experimental runs.

## 1.12 Modeling of ATP binding

The web-based tool ATPint^27^, was used to predict ATP binding residues on the surface of rabbit fast skeletal myosin 2 (PDB: 5H53) or BSA (PDB: 3V03). This tool relies on a Support Vector Machine (SVM) based model trained on non-redundant ATP-binding sequences and other information^27^. The model gives a score as output in the range -1.0 to 1.0 for each amino acid residue. In order to assign a residue as ATP-binding, a threshold must be set by the user. Here, a threshold of 0 suggests similar sensitivity and specificity of the assignment whereas a value>0 suggests higher specificity (few erroneously classified ATP binding sites) than sensitivity (with increased risk of missing real binding sites). In our analyses we used the threshold 0.2, unless otherwise stated.

The ATP binding residues were plotted using PyMOLmolecular graphic system (ver. 1.2r3pre)^28^. In addition to ATPint we have also tested other ATP binding-prediction software (e.g. ATPbind^29^, TargetATPsite^30^, IBIS^31^). Most tools find the active site ATP binding as well as some outside residues (these differed between tools). ATPint, however, finds all the residues outside the active site found by other tools. Finally, supported by the fact that ATPint could predict nonspecific ATP binding to BSA^32,33^ we selected this software for further use.

# 2. Supplementary Results and Discussion

## 2.1 Effects of different laser power

A straightforward approach that is often used^21^ to distinguish dye photophysics events (bleaching and blinking) from actual enzymatic reactions is to change the illumination intensity and check if kinetic rate constants or any relative population amplitude changes. Ideally, the experimental setup is designed so that the illumination intensity at each step is never so high that the photobleaching phenomena overwhelm the system. Assuming this is the case, one would expect that with increasing illumination intensity the kinetics originating from blinking gets faster and higher in relative amplitude, while the kinetics of enzymatic reactions do not change. However, results in the literature^34^ imply that effects of altered illumination intensity on blinking time and blinking fraction vary between fluorophores and experimental conditions, despite the fact that that it is usually assumed that higher laser power yields higher blinking fraction (e.g. ^35^). Experimental results at varied illumination intensity need to be viewed against this background. Nevertheless, during our methods developments (buffer compositions, myosin depositions, and coverslip cleaning method) we have frequently performed assays at two different laser powers of ~0.7 and ~2.7 mW (as measured at the back focal plane); technical limitations prevented us from extending this range. Generally, our results (Fig. S3) showed no consistent effects of varied illumination intensity on the rate constants and fractional amplitudes of different exponential phases in the Alexa-ATP on-dwell-time distributions (Fig. S3). In summary, the results suggest that the photophysics (photoblinking in particular) of the Alexa647 fluorophore does not change appreciably with changes in illumination intensity in the range and under the conditions tested. This somewhat unexpected behavior can be related to a recent finding reporting that the origin of Alexa647 dye blinking may be rather complex involving Alexa647 reversible photo-induced isomerization to at least two long-lived dark species in addition to the traditionally accepted importance of triplet excited states^36^. Under certain experimental conditions (i.e. buffer compositions) this could further obscure the mechanism of the blinking process itself and its laser intensity dependence.

**Fig. S3. Dependence of the processes detected in cumulative frequency distribution of Alexa647-nucleotide dwell-time events on the laser power (in milliwatts, mW).** **a** Data collected by using simple myosin deposition and IVMA assay buffer were best fitted to double exponential function. Data for low laser power replotted from Fig. 2f. **b** Amplitudes and rate constants obtained from the fitting of data under a. **c** Data collected by using simple myosin deposition and IVMA assay buffer supplemented by 2mM of not optimized TX/TQ (TX/TQ = 9%) were best fitted to triple exponential function. **d** Amplitudes and rate constants obtained from the fitting of data under c. Note, that unlike in b, a slow phase consistent with k_cat_ from solution can be identified. **e** Data collected by using simple myosin deposition and optimized assay buffer A130 (ionic strength 130 mM) were best fitted to double exponential function. **f** Amplitudes and rate constants obtained from the fitting of data under e. **g** Data collected using optimized myosin deposition and optimized assay buffer A60 were best fitted to double exponential function. **h** Amplitudes and rate constants obtained from the fitting of data under e. Note: no clear and consistent effect of almost 4-fold change in laser power on the distribution of rates and amplitudes of detected processes under different experimental conditions. In panels b, d, f and h, error estimates refer to 95 % confidence intervals derived in the regression analysis. See further SI text section 2.1.

## 2.2 Optimizing the assay buffer for TIRF experiments

A cocktail of triple state quenchers (Trolox, COT, NBA) have been used before to stabilize the Alexa647 fluorophore in a context-dependent manner i.e. to provide photostabilization in different dye microenvironments^37^. To the best of our knowledge, however, none of these triple state quenchers have previously been used for single molecule assays of myosin or actomyosin^16^. Since it is known that each fluorophore may require its own triple state quencher(s) and that the microenvironment can influence fluorophore photophysics^38^ we first investigated how the different triple state quenchers affect Alexa647 in the context of myosin and actin. As can be seen from Fig. S4c, the combination of all triple state quenchers was most effective. As a novelty of this study, we also optimized the ratio between Trolox-Trolox and Trolox-Quinone with respect to the effectiveness in combating photophysical complications (Fig. S4e-f). In the process of the optimization, we exposed the Trolox solution to UV light for different times^1,39^, measured the Trolox-Trolox and Trolox-Quinone fractions spectrophotometrically (Fig. S4e)^1^ and then tested the mixture in the single molecule assay. This analysis (Fig. S4e-g) suggested an optimal Trolox-Trolox/Trolox-Quinone ratio of ~0.2 that was subsequently used unless otherwise stated.


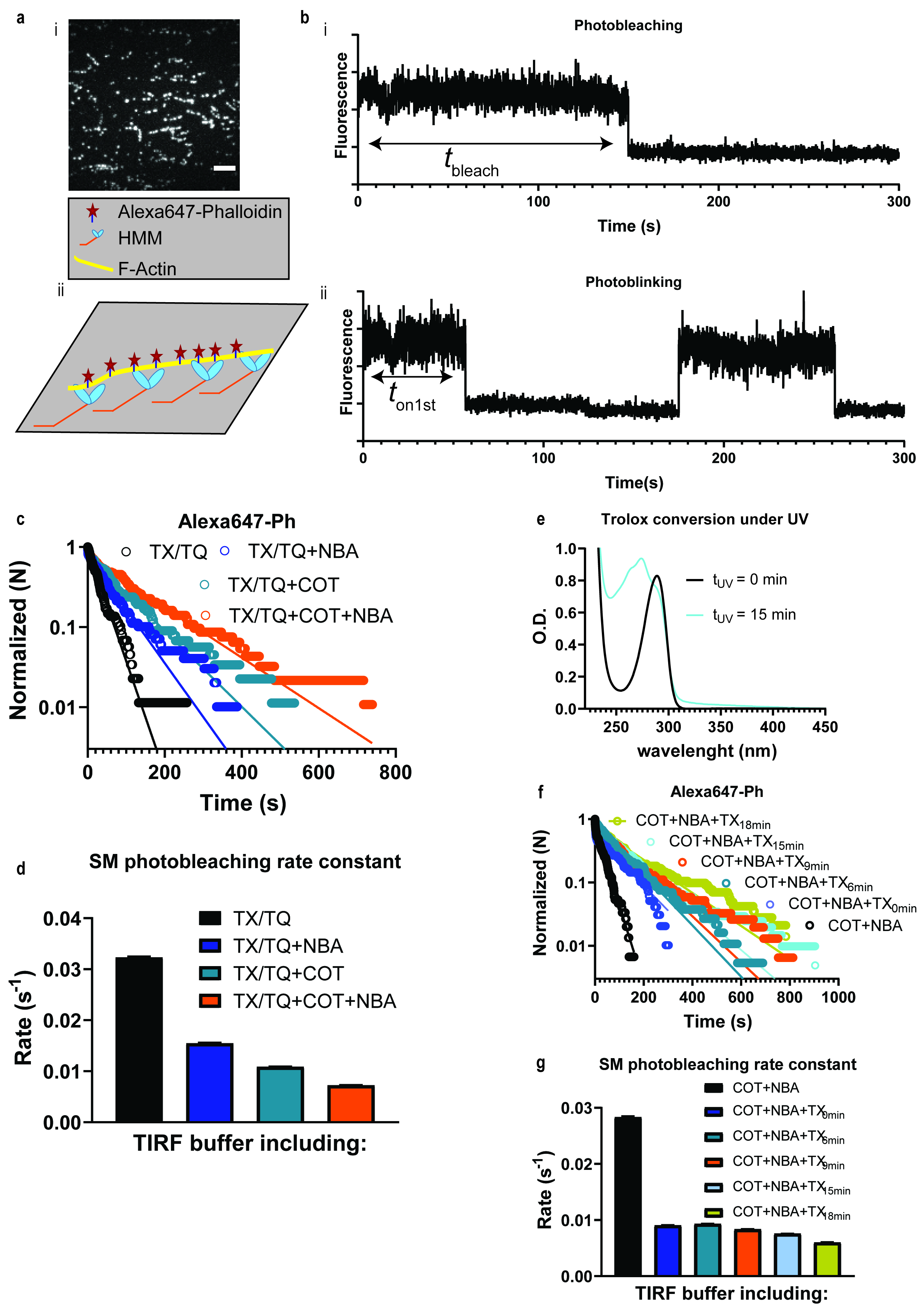


**Fig. S4. Stabilizing Alexa647 dye photophysics. a** Image (i) and schematic presentation (ii) of F-actin filaments partly labelled (1:240 molar ratio) with Alexa647-Ph, which was used in these experiments to uncouple fluorescence change due to ATPase activity and photobleaching/blinking. Bar, 5 µm. **b** Representative time traces of single molecule photobleaching (i) and photoblinking (ii) of Alexa647-Ph. Photobleaching times (t_bleach_) or times until first photoblinking event (t_on1st_) were measured and used in further analysis below. **c** Cumulative frequency distribution of single molecule Alexa647 t_bleach_ and t_on1st_ in TIRF buffer containing different triple state quenchers and redox active components: Trolox-Trolox/Trolox-Quinone (TX/TQ), 4-Nitrobenzyl alcohol (NBA), Cyclooctatetraene (COT). Note appreciable increase in t_bleach_ or t_on1st_ when all three components were included. The distributions are satisfactorily fitted by a single exponential function (solid lines). **d** Single molecule photo-bleaching (-blinking) rate constants obtained from the fitting of data in c. Error estimates refer to 95 % confidence intervals derived in the regression analysis. Note appreciable reduction of rate constant values by performing experiments in TIRF buffer containing all three components. **e** TX conversion to TX/TQ under exposure to UV light as observed by difference in absorbance spectra^1^. **f** Cumulative frequency distribution of single molecule Alexa647 t_bleach_ and t_on1st_ in TIRF buffer containing NBA and COT which was further supplemented by TX/TQ with varying amount of TQ achieved by exposing the TX solution to different amounts of UV radiation (in min). Note appreciable increase in t_bleach_ or t_on1st_ when [TQ]/[TX] reaches ~ 0.2 at 15 min UV exposure. The distributions are well fitted with a single exponential function (solid lines). **g** Single molecule photo-bleaching (blinking) rate constants obtained from the fitting of data in c. Error estimates refer to 95 % confidence intervals derived in the regression analysis. Note appreciable reduction of rate constant values by performing experiments in TIRF buffer containing all three components at higher [TQ]. We used 15 min UV exposure ([TQ]/[TX] ~ 0.2) for all experiments unless otherwise stated. See further SI text section 2.2.

We also examined the possibility that different fluorophore microenvironments of Alexa-ATP in the myosin active site and Alexa-Phalloidin on actin may affect photobleaching/blinking. The studies of Alexa-nucleotide in the active site were performed by first forming a stable myosin·Alexa-ADP·Vi complex^40,41^ to allow studies of blinking/bleaching without interference from turnover events. Although the bleaching rate constants were comparable between Alexa-Phalloidin and Alexa-ATP/ADP locked in the myosin active site pocket, extra viscosity was needed (realized by adding methylcellulose) to stabilize the fluorescence signal (Fig. S5).


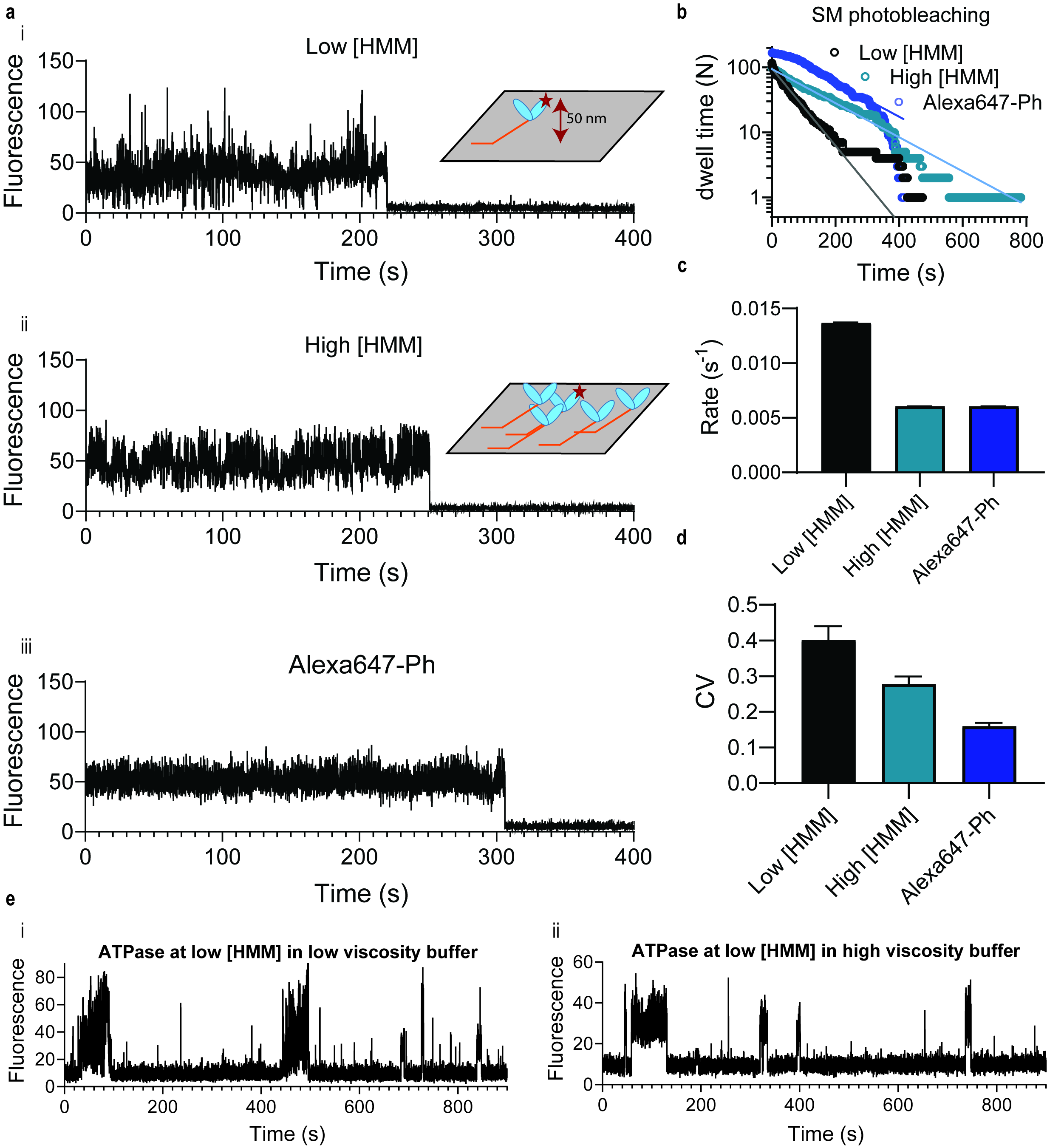


**Fig. S5. Stabilizing the Alexa647 dye signal.** **a** Representative time traces for single molecule photobleaching of Alexa647-ADP locked with vanadate into the nucleotide pocket (so called HMM*D·Vi complex) at (i) low [HMM*D·Vi], (ii) at low [HMM*D·Vi] supported with saturated concentration of nucleotide-free HMM, (iii) Alexa647-Ph (as in Fig. S4). **b** Cumulative frequency distribution of single molecule Alexa647 photobleaching under experimental conditions presented in a. The distributions can be fitted with a single exponential function (solid lines). **c** Single molecule photo-bleaching rate constants obtained from the fitting of data in b. Error estimates, 95 % confidence intervals derived in the fits. **d** Signal stability estimated by calculation of signal CV. Note appreciable reduction of CV when HMM*D·Vi was stabilized by other HMM molecules or when observing the fluorophore firmly attached via phalloidin to the actin filament. Appreciable noisiness of the signal in the case of low [HMM*D·Vi] may be attributed to thermal fluctuations of HMM. Such fluctuations (~ 50 nm up and down in total, see^16^) would be sufficient under evanescent wave TIRF illumination to cause considerable fluctuations of the fluorescence signal. **e** Representative time traces of single molecule HMM ATPase performed in TIRF buffer with (i) or without (ii) methylcellulose. High viscosity of the buffer damped thermal fluctuation of HMM, improving overall signal stability. See further SI text section 2.2.

## 2.3 Specific versus nonspecific binding of Alexa647–ATP and Alexa647 moiety to assay surface.

By use of extensive surface cleaning, optimized selection of BSA and deposition of myosin via actin (main Fig. 2a ii-iii) we have greatly minimized the risk of artefacts due to dwell time events not associated with myosin motor fragments. However, with the aim to fully eliminate this risk we compared the intensity–time traces of regions of interest (3 × 3 pixels) *outside “hotspots*” - that is, outside regions of 3 × 3 pixels with many repetitive binding events - versus intensity–time traces of “*hotspots” themselves*. From comparison of the traces and the collected dwell times we estimated that, on average 2 ± 1 (mean ±SD, N=168 traces from 5 independent experiments) dwell times appear in a region of interest of 3 × 3 pixels throughout a total observation period (15 min) in the absence of myosin. The plotted cumulative dwell time distributions for out-of-hotspot events were differing greatly from the distributions for the hotspots (Fig. S6). For control purposes (Fig. S6c), we subtracted out-of-hotspot events from hotspot events. This was done by arranging the events from background and hotspots into two histograms with bins equal to exposure time (52 ms) followed by bin wise subtraction (Hist_hotspots_-Hist_background_). Fitting of the corrected cumulative dwell time distribution from hotspots yielded rates and amplitudes essentially equal to those obtained from uncorrected dwell time distributions (Fig. S6a-c). This confirms that nonspecific Alexa647-ATP binding to the surface (BSA or the underlying surface), under our experimental conditions is negligible. Furthermore, in order to examine any unspecific binding of the Alexa647 moiety of Alexa-ATP, we utilized Alexa647-cadaverine. From the results of binding experiments, designed as for the true ATPase studies except for replacing Alexa-ATP by Alexa647-cadaverine, we conclude that the Alexa647 moiety does not play any role in Alexa-ATP binding events (Fig. S6d-e).


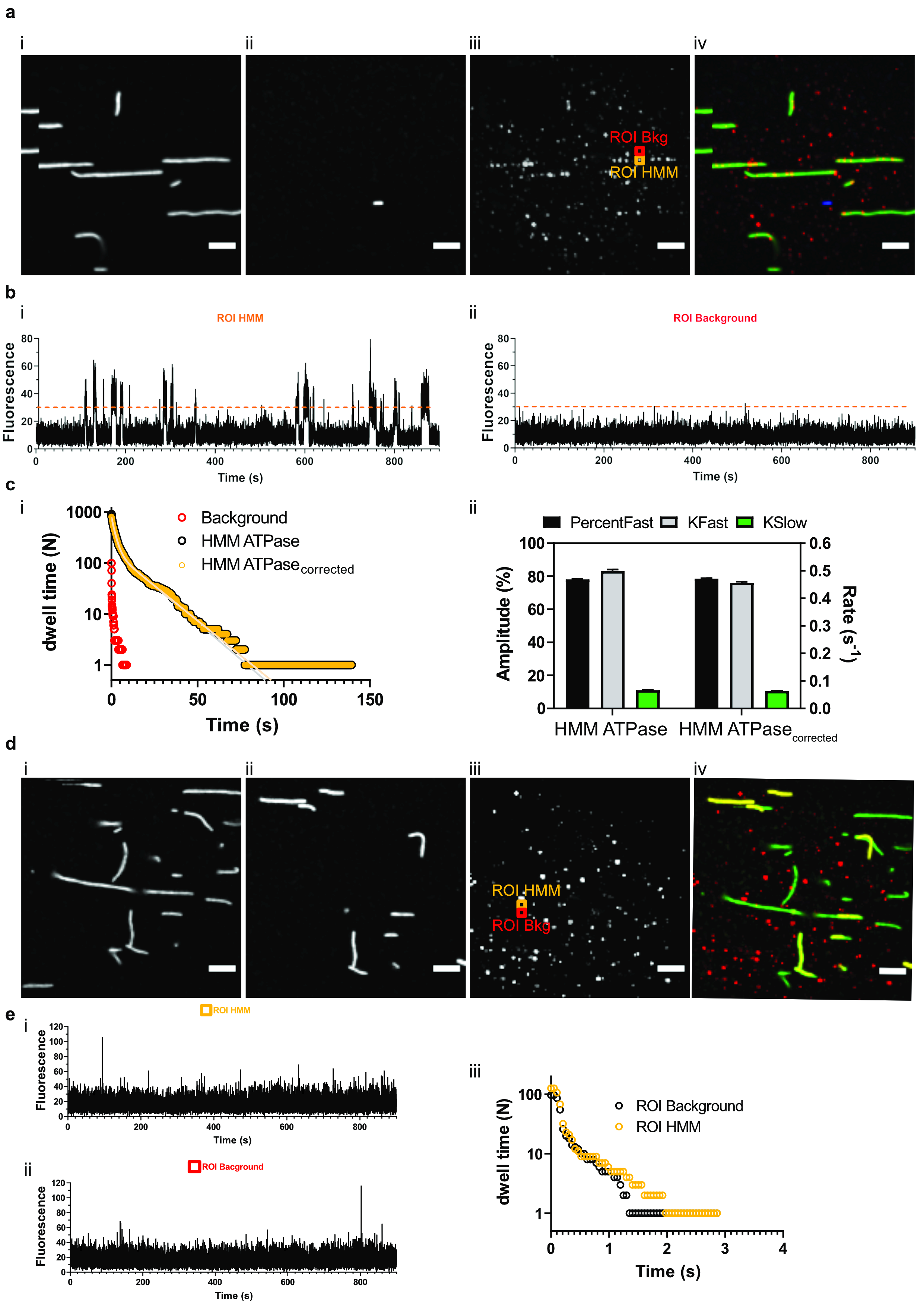


**Fig. S6. Specific vs unspecific binding of Alexa-ATP and Alexa647 moiety (using Alexa647-cadaverine). a** Figure set depicting optimized HMM deposition with Rhodamine phalloidin labeled actin filaments before (i) and after (ii) high [ATP] = 100 µM wash together with (iii) Alexa-ATP time projection fluorescence (see text for definition) of 15 min videos (50 ms exposure time/frame). Only spots colocalized with washed-away F-actin filaments were included in the analysis (iv, green - F-actin filaments before ATP wash, blue - F-actin filament after ATP wash, red - time projected Alexa-ATP binding events). Bars, 5 µm. **b** Representative time traces of “ROI HMM” (see a, iii) depicting HMM basal ATPase and “ROI Background” (“ROI-Bkg”, see a, iii) depicting any unspecific Alexa-ATP binding in immediate vicinity of the HMM location. The dashed line presents the same threshold used in both traces to collect dwell time events in a consistent manner. **c** Cumulative frequency distribution of Alexa-nucleotide dwell-time events in background, HMM surface hot-spots, and HMM background-corrected hotspot (see also text). The latter two were well fitted with double exponential functions (solid lines). HMM ATPase data from 33 HMM molecules, N_dwell_ = 911, were corrected using 33 background traces with N_dwell-background_ = 99. After subtraction, the HMM ATPase_corrected_ had N_dwell_ = 813. Note, no apparent difference in rates and amplitudes (ii) after HMM hotspots were background corrected. Error estimates in panel c(ii), 95 % confidence intervals derived in the fits. **d** Figure set examining nonspecific binding of Alexa647-cadaverine not expected to bind to the myosin active site. Optimized HMM deposition with Rhodamine phalloidin labeled actin filaments before (i) and after (ii) high [ATP] = 100 µM wash together with (iii) Alexa647-cadverine time projection fluorescence of 15 min videos (50 ms exposure time/frame). Only ROIs colocalized with washed-away F-actin filaments were included in the analysis (iii, green - F-actin filaments before ATP wash, yellow - F-actin filament after ATP wash, red - time projection of Alexa647-cadaverine binding events). Bars, 5 µm. **e** Representative time traces of (i) “ROI HMM” (from d, iii) depicting events on what seems to be HMM hotspots after adding Alexa647-cadaverine and (ii) “ROI Bkg” (see also d, iii) depicting events in immediate vicinity of the presumed HMM hotspot. Note (iii) no apparent difference in dwell time distributions for events associated with actin filament (“ROI-HMM”) and not (“ROI-Bkg”). In total 58 ROIs were analyzed: 29 ROI HMM, 29 ROI Background producing 126 and 99 dwell times, respectively, similar to non-specific binding of Alexa-ATP outside hotspots (see **c** above). See further SI text section 2.3.

## 2.4 S1 and HMM ATPase under optimized conditions

We used the optimized assay conditions (cf. summary section under Materials and Methods) to study single molecule S1 (simple deposition method) and HMM (optimized myosin deposition) basal ATPase (Fig S7 and Fig 2 in main paper). In all cases, there was a slow phase consistent with basal MgATP turnover by myosin. An almost 10-fold faster unexplained phase was also invariably present. The results were quantitatively similar in two experiments using S1 and two experiments using HMM. This demonstrates the reproducibility of the assay as well as the negligible complications introduced by the two-headed nature of HMM under our optimized assay conditions.

**Fig. S7**. **Reproducibility of optimized single molecule ATPase assay.** Left: Cumulative frequency distributions of Alexa-nucleotide dwell-time events comparing HMM and S1 basal ATPase activity under optimized conditions on four different experimental occasions. The distributions are reasonably well fitted with double exponential functions (solid lines). Right: Amplitudes and rate constants obtained from the fitting of data on the left. Columns represent means and error estimates refer to 95 % confidence intervals derived in the regression analysis. Temperature: 23 °C. See further SI text section 2.4 and Fig. 2 in main paper (HMM_1_ replotted from Fig. 2).

**Fig. S8. Double-exponential vs Triple exponential fit to cumulative frequency distributions for Alexa-nucleotide on-time events.** Left: Cumulative frequency distributions of Alexa-nucleotide dwell-time events comparing HMM and S1 basal ATPase activity under optimized conditions. The distributions are fitted with double (solid lines) or triple (dashed lines) exponential functions. Right: Amplitudes and rate constants obtained from the fitting of data on the left using triple exponential function. Bar heights represent mean values and error estimates refer to 95 % confidence intervals. Note, the “fast phase” corresponds to the unexplained phase in double exponential fittings whereas the phase attributed to ATP turnover in double-exponential fits is here subdivided into a “medium” (0.05-0.1 s^-1^) and “slow” (<0.05 s^-1^) phase. Error estimates, 95 % confidence intervals derived in the fits. Temperature: 23 °C. Qualities of the fits and further details in Table S1. Same data as in Fig. S7. See further SI text section 2.4 and Fig. 2 in main paper (HMM_1_ replotted from Fig. 2).

**Table S1: Summary of best mean parameter values in double, triple, or triple with weighting (1/time) exponential fits. For 95 % CI estimation please see corresponding figures (Fig. S7, Fig S8):**

| ATPase | *k*_fast_ (s^-1^) *A*_fast_ (%) | *k*_inter_ (s^-1^) *A*_inter_ (%) | *k*_slow_ (s^-1^) *A*_slow_ (%) | *r*^2^ | AICc |
| --- | --- | --- | --- | --- | --- |
| HMM_1_, N=785 *triple exp.* | 0.56 51.5 | 0.11  35.8 | 0.038  12.7 | 0.9995 | 3782 |
| *^a^ triple exp  weight 1/t.* | 0.3773  ~ 10.91 | ~ 0.0586  ~78.18 | ~ 0.0583  ~ 10.91 | 0.9992 | 5233 |
| *double exp.* | 0.43  63.29 | 0.063  36.71 |  | 0.9985 | 6064 |
| HMM_2_, N=387  *triple exp.* | 0.44 52.2 | 0.09  40.8 | 0.022 7.0 | 0.9991 | 2580 |
| *^a^ triple exp  weight 1/t.* | 0.275  70.9 | 0.048  28.6 | ~ 0  0.05 | 0.9987 | 3789 |
| *double exp.* | 0.31  68.18 | 0.045  31.82 | / | 0.9971 | 6046 |
| S1_1_, N= 345  *^a^ triple exp.* | 0.25  66.95 | 0.05  32.90 | ~ 4.930e-032  0.15 | 0.9984 | 4037 |
| *^a^ triple exp.  weight 1/t* | n.a. | n.a. | n.a. | n.a. | n.a. |
| *double exp.* | 0.25  67.98 | 0.048  32.02 | / | 0.9983 | 4150 |
| S1_2_, N=1964  *triple exp.* | 0.37 35.0 | 0.071  59.5 | 0.019  5.6 | 0.9998 | 10436 |
| *triple exp.  weight 1/t* | 1.1  (1.0 to 1.2)^b^  11.4  (10.5 to 12.3)^b^ | 0.20  (0.19 to 0.21)^b^  43.6 | 0.05  0.049 to 0.051^b^  45  (44 to 47)^b^ | 0.9998 | 9810 |
| *double exp.* | 0.24  49.89 | 0.050  50.11 | / | 0.9989 | 18320 |

^a^ fitting did not converge properly or was ambiguous

^b^ 95 % CI

Since the fastest phase (rate constant >2 s^-1^), ubiquitous and dominant in a previous study^19^ was completely lost we tried to fit the data from Fig. S7 with triple exponential functions in order to potentially resurrect this phase. As can be seen from Fig. S8 triple exponential fits did not achieve this, despite setting initial values in the regression procedure such that the rate constants were similar to the fastest ones seen previously. Rather an extra slow phase emerged but with quite small amplitude.

Notably, however, due to the use of cumulative distribution plots, the data are dominated by the shortest events and with increasing interdependence of the data with increasing time. In order to investigate if this fact erroneously eliminates a dominating fast phase in the non-linear regression fit, we also fitted the data with increased weight (proportional to 1/time) for short times. Strikingly, however, for majority of these cases fitting was ambiguous and complete confidence intervals could not be calculated (Table S1). An exception was the S1 ATPase2 where fitting was successful, showing a fast phase with *k*_fast_ = ~1.1 s^-1^ (~11 %). It seems that when enough dwell times are collected (in this case almost 2000) and fitting is weighted (1/time), a faster phase can be discerned. However, importantly, the latter phase is of low amplitude, far from the dominating role in previous work (and our experiments in the IVMA solution; Fig. 2 in main paper). Furthermore, the rate constant is less than half of that found previously and not more than about twice that of our unexplained phase (0.2-0.5 s^-1^). Neither was the fast phase resurrected by limiting the fitting to times <10 s without weighting.

We also used the optimized assay conditions to study single molecule HMM-actin-activated ATPase (Fig. 2g-i; Fig S9). For both dwell-time data-sets triple exponential functions gave good fits with the fastest rate constant attributed to actin-activated ATP turnover.

**Fig. S9.** **Reproducibility of optimized single molecule actomyosin ATPase assay. a** Cumulative frequency distribution of Alexa-nucleotide dwell-time events of actomyosin ATPase activity under optimized conditions. Data from 36 actomyosin hotspots, N_dwell_ = 1290. The distributions are fitted by a triple exponential function (solid line). **b** Amplitudes and rate constants obtained from the fitting of data on the left. Note that the values are similar to the actomyosin data in main Fig 2 but they refer to other experiments. Columns represent means and error estimates refer to 95 % confidence intervals derived in the fits. Temperature: 20 °C. See further SI text section 2.4 and Fig. 2 in main paper.

## 2.5 Effects of Alexa-ADP

One possible basis for the unexplained phase (0.2-0.5 s^-1^) is that it is attributable to rebinding of Alexa-ADP. However, if that had been the case its amplitude would increase over the period of observation (15 min) or new very fast phases would be expected to emerge. To test the possibility of Alexa-ADP accumulation we subdivided the 15 min traces into three equal parts (beginning, middle, end) and analyzed them separately. As can be seen from Fig. S10 there was no clear trend to suggest increased amplitude or rate of the fast, unexplained phase over time that might suggest Alexa-ADP accumulation.


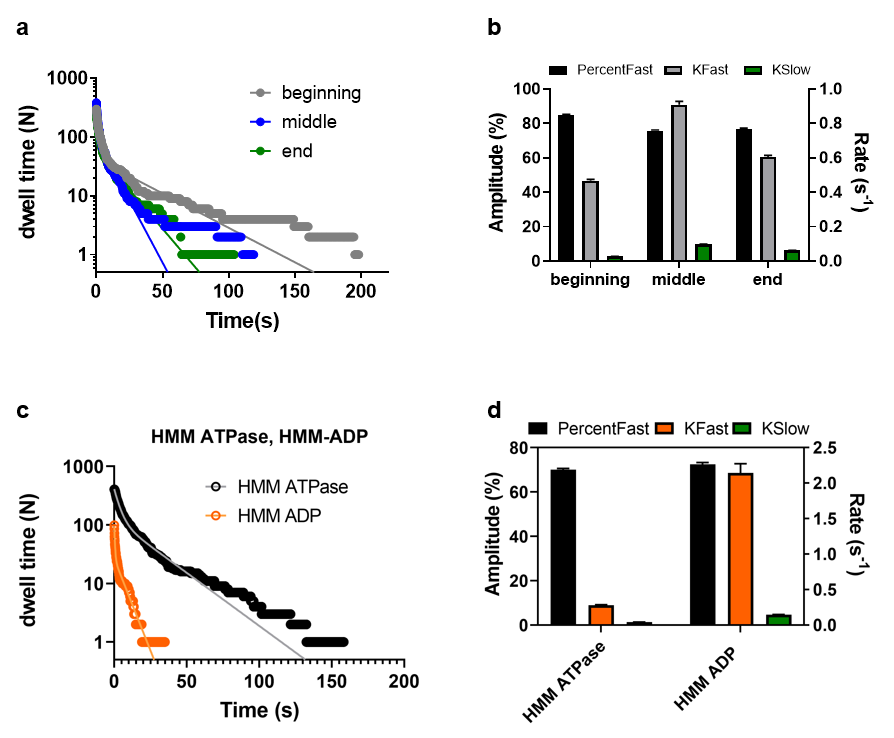
**Fig. S10.** **Single molecule HMM basal ATPase: role of Alexa-ADP.** **a**: Cumulative frequency distribution of Alexa647-nucleotide dwell-time events comparing beginning, middle and end part of the 15 min traces. The distributions were well fitted with double exponential functions (solid lines). **b**: Amplitudes and rate constants obtained from the fitting of data to the left. **c**: Cumulative frequency distribution of Alexa647-nucleotide dwell-time events comparing regular HMM ATPase vs events distribution under “ADP conditions” (see text). **d:** Amplitudes and rate constants obtained from the fitting of data on the left. Columns represent means and error estimates refer to 95 % confidence intervals derived in the fits. Temperature: 23 °C. See SI text section 2.5. HMM ATPase reused from Fig. S7.

To further address the possible contribution of Alexa-ADP binding we created “Alexa-ADP” conditions by pre-incubating HMM with AlexaATP in wash buffer for 1-2 h at room temperature. The reaction mixture was then diluted in TIRF buffer and dwell time events were directly observed. Analysis of extracted traces showed dwell-time distributions with hugely different appearance than in studies of basal HMM ATPase. Particularly, there was an extra dominating fast phase (~2s^-1^) with about 20-fold faster rate than basal HMM ATPase and more than 4-fold faster rate than the unexplained phase in HMM ATPase (Fig. S10). This further substantiates our conclusion that the unexplained phase observed in basal HMM ATPase is not attributable to (re)binding of Alexa-ADP.

## 2.6 Blocking the myosin active site by fluorescent Alexa-ATP in the presence of vanadate.

A modified version of the experiment in Fig. 5 (main text) was performed where Alexa-ADP (instead of non-fluorescent ADP) was locked to the myosin active site in the presence of vanadate, forming a fluorescent S1*aD·Vi complex. Subsequently the experiments were performed as in Fig. 5 by adding 10 nM Alexa-ATP to the assay buffer. The Alexa-ATP binding in this case led to fluorescence intensity events that were superimposed on top of those attributed to Alexa-ADP locked by the vanadate at the active site (Fig. S11). The analysis of only the latter double-intensity events, showed (Fig. S12) closely similar distributions for Alexa-ATP *on* dwell times as when non-fluorescent ADP was locked to the active site (Fig. 5).

**Fig. S11. Representative time traces of Alexa-ATP binding to fluorescent S1*aD·Vi complex hotspot consistent with unspecific Alexa-ATP binding to myosin outside the active site.** F(S1*aD·Vi): long fluorescent dwell times representing bleaching-limited fluorescence of S1*aD·Vi complex. F(aT|S1 S1*aD·Vi): short fluorescence dwell times on top of longer one, attributed to unspecific interaction of Alexa-ATP with S1 myosin outside the active site. See further SI text section 2.6.

**Fig. S12. Supportive data for Fig. 5 (main text) to determine the origin of “unexplained” (0.2-0.5 s^-1^) exponential phase. a** Triplicate of cumulative frequency distributions of Alexa-nucleotide dwell-time events (see Fig. S11) on myosin subfragment 1 (S1) surface hotspots (simple deposition of S1, Fig. 2a, sub-panel i). Data from 36 (#1), 25 (#2), 29 (#3) S1*aD·V_i_ hotspots where nucleotide pocket was blocked by fluorescent Alexa-ADP and Vanadate. The data were fitted by double exponential functions (solid lines). **b** Amplitudes and rate constants obtained from fittings to data to the left are comparable to the data when non-fluorescent ATP was used to block S1 active site (Fig. 5, main text). Notably basal ATPase activity (k_ATPase1_ ~ 0.05 s ^-1^) is here not detected, consistent with the fact that we analyzed only events on top of prolonged fluorescent signals attributed to the S1*aD·V_i_ complex (i.e. S1 with blocked active site). Error estimates refer to 95 % confidence intervals obtained in the fits. Temperature: 23 °C. See further SI text section 2.6.

## 2.7 Equilibrium dialysis experiments

Orthogonal evidence for Alexa-ATP binding outside the active site of myosin is provided by equilibrium dialysis experiments (Figs. S13-S14). In these experiments, a semipermeable membrane separates the ligand, (Alexa-ATP), from the macromolecule (HMM) and the samples are allowed sufficient time to equilibrate (overnight at 4 °C). The concentrations of ligand after equilibration in the ligand-only chambers dialyzed against HMM or HMM-free (control) chambers, were then used to calculate the number of nucleotides bound to HMM. The experiments were performed at two HMM concentrations (~0.3 µM, ~1.1 µM) and three ionic strength buffers of 20, 60 and 130 mM (W20, W60, E130). Experiments at low [HMM] were performed at constant ionic strength of 60 mM (W60) similar to the ionic strength in a majority of the TIRF assays. Lower [HMM] was chosen in order to perform experiments at greater excess of Alexa-ATP. By that we should in principle be able to examine full saturation of all ligand binding sites with the commercially available Alexa-ATP stock concentration. The other reason was to minimize HMM consumption. These experiments were, however, challenging due to noisy absorbance spectrometry data with low difference in absorbance between HMM and HMM-free runs combined with the above addressed nucleotide loss and imbalance (Fig. S13).

**Fig. S13. Calibration of the equilibrium dialysis experiments.** **a** absorbance spectra of diluted initial [ATP] sample (divided by 2, theoretical equilibrium value) and final diluted [ATP] sample from initially ATP-full chamber 1 and ATP-empty chamber 2 performed in LISS buffer. **b** absorbance spectra of diluted initial [Alexa-ATP] sample (divided by 2, theoretical equilibrium value) and final diluted [Alexa-ATP] sample from initially Alexa-ATP-full chamber 1 and Alexa-ATP-empty chamber 2 performed in LISS buffer. **c** the same as b but performed 130 mM ionic strength buffer (W130) buffer. Note that nucleotide loss (0.1 ± 1.6 %, N=5, mean + SD) and imbalance between the chambers (0.03 ± 2.9 %, N=5, mean + SD) was as expected negligible with ATP as a ligand but more substantial with Alexa-ATP in LISS buffer (loss: 2.2-5.2 %, imbalance: 61-75 %) with less imbalance in high ionic strength buffer W130 (loss: 9.1 ± 2.9 %, imbalance: 2.8 ± 2.5 %, N=3, mean + SD). See further SI text section 2.7.

Despite the challenges, we found evidence for unspecific nucleotide binding sites outside the active site. Thus, at low [Alexa-ATP] (<5 µM) HMM binds ~2 nucleotides/HMM, i.e. one on each head (Fig. S14). This is consistent with expected catalytic site binding for the case with negligible amount of non-functional heads. The extra binding sites detected at higher [Alexa-ATP] must then represent binding to additional unspecific ATP binding sites. We found evidence for up to ~ 9 binding sites per HMM molecule (i.e. ~4 per myosin head; Fig. S14c) and we observed binding to more than two sites at [Alexa-ATP] below 10 µM (Fig. S14c). Overall, the results are consistent with the existence of up to 4 unspecific nucleotide binding sites outside the myosin active sites with association constant for the most strongly binding sites in the micromolar range as further elaborated on in the main paper.

**Fig. S14: Equilibrium dialysis experiments.** **a** Absorbance spectra of 20x diluted final post-equilibrium dialysis [Alexa-ATP] in nucleotide-only chamber dialyzed against high ionic strength (130 mM, W130) buffer only (control, [aT]_f, HMM-free_) or HMM sample in that buffer ([aT]_f, HMM_, [HMM] =1.02 µM) at initial, pre-equilibrium [Alexa-ATP] = 10 µM. The net change in the absorbance indicates ~1.7 nucleotide binding sites per HMM in this specific experiment. **b** The same as a, but at [HMM] =1.24 µM and at initial, pre-equilibrium [Alexa-ATP] = 100 µM. The net change in the absorption indicates ~5.7 nucleotide binding sites per HMM. Note, different scale on absorbance axis compared to a. Inset: top of the curves shown on similar scale as in a. **c** Number of nucleotides per HMM molecule measured and analyzed as in a and b for different experimental conditions as indicated. Data represent mean ± SD with N=1-3. Data from W60 conditions were fitted to simple rectangular hyperbola (blue solid line) to guide the eye. The fit is saturating at 9 bound nucleotides (6-12; 95% CI). Dashed black line represents maximal expected nucleotides bound per HMM molecule if there is binding only to the active sites and if all HMM molecules are functional. Not that the lower values obtained at 1.1. µM HMM are partly attributed to almost four-fold less excess of free Alexa-ATP. These data were nevertheless included due to higher signal/noise ratio in the spectrophotometric analysis. See further SI text section 2.7.

**Fig. S15. Effect of assay buffer ionic strength on observed processes in TIRF based basal myosin ATPase assay. a, c** Cumulative frequency distributions of Alexa-nucleotide dwell-time events comparing HMM basal ATPase activity in assay (A) buffers with different ionic strength, 20, 60 and 130 mM, from two experiments. The data were fitted by double exponential functions (solid lines). **b, d** Amplitudes and rate constants obtained from fittings to data in a and c. Columns represent means and error estimates refer to 95 % confidence intervals derived in the fits. Temperature: 23 °C. See further SI text section 2.8.

## 2.8 Effect of ionic strength on the Alexa-ATP on-dwell time distributions

In order to shed more light on the nature of the non-specific Alexa-ATP interactions we performed experiments at different ionic strengths (Fig. S15). These experiments showed inconclusive effects of varied ionic strength on the amplitudes and rates of the unexplained phase; though with certain variability of the rate at highest ionic strength studied. These findings are consistent with the idea that the non-specific binding events involve both ionic and non-ionic interactions (because higher ionic strength contributes to stronger hydrophobic interactions but weaker electrostatic interactions^42,43^). This is in accordance with the ATPint modelling data (section 2.9 below) suggesting that amino acids with both basic (16 %) and hydrophobic (46 %) side-chains are involved in the areas with predicted binding.

## 2.9 Modeling of ATP binding

We used an on-line bioinformatics tool to investigate the possibility of further binding sites for ATP on the surface of myosin outside the active site. Several online engines where tested and compared (ATPbind^29^, Target ATPsite^30^, ATPint^27^, IBIS^44,45^, FTmap^46^ and NsitePred^47^). To check the efficiency of the search parameters of each engine, the control protein BSA was used. BSA has been found to have weak surface interactions with ATP^32,33^ and is therefore appropriate reference for sensitivity to non-specific binding. All the chosen search engines where able to correctly identify the active site on myosin. However, the ATPint software, unlike the others, stood out by identifying several additional spots on the surface of both BSA and myosin to be able to bind ATP. We further examined the ATPint software threshold parameter. Results from different ATPint thresholds on detection of binding residues are presented below and visualized in Fig. S16 for the 5H53 sequence (rabbit skeletal muscle myosin II in rigor) and 3V03 (BSA). Cumulative data for thresholds **0.0 0.2 0.6 1.0** (note: threshold =1 has the least residues and threshold =0.0 has the most; the negative values for the threshold are not useful, just blurring all residues).

In conclusion, after testing several thresholds (see above), we decided to choose 0.2 because it identified multiple interacting patches and not only individual residues on the surface of BSA. Furthermore, on myosin, use of the threshold 0.2 lead to identification of all of the residues outside of the active site that are indicated by combining the results of the other software (Table S2).

**Table S2: Comparison of different tools to detect ATP binding sites**

| **Tool** | Sequence  (PDB) | Protein name | Residue  (total#) | Binding residues (%) | Basic  (%) | Hydrophobic  (%) |
| --- | --- | --- | --- | --- | --- | --- |
| ATPbind | 3V03 | BSA | 583 | None detected | | |
|  | 5H53 | Myosin | 845 | 2.0 | 11.7 | 23.5 |
| Target ATPsite  (0.7) | 3V03 | BSA | 583 | None detected | | |
|  | 5H53 | Myosin | 845 | 1.5 | 7.7 | 23 |
| ATPint (0.2) | 3V03 | BSA | 583 | 13.2 | 19.5 | 31.2 |
|  | 5H53 | Myosin | 845 | 30.5 | 16 | 46 |
| IBIS | 3V03 | BSA | 583 | None detected | | |
|  | 5H53 | Myosin | 845 | 2.1 | 5.5 | 27.8 |
| FTmap  *(Not limited to binding of ATP!)* | 3V03 | BSA | 583 | 19.9 | 19.8 | 48.3 |
|  | 5H53 | Myosin | 845 | 15.3 | 9.3 | 45 |
| NsitePred (ATP, ADP, AMP data) | 3V03 | BSA | 583 | None detected | | |
|  | 5H53 | Myosin | 845 | 3.8 | 31.2 | 15.6 |


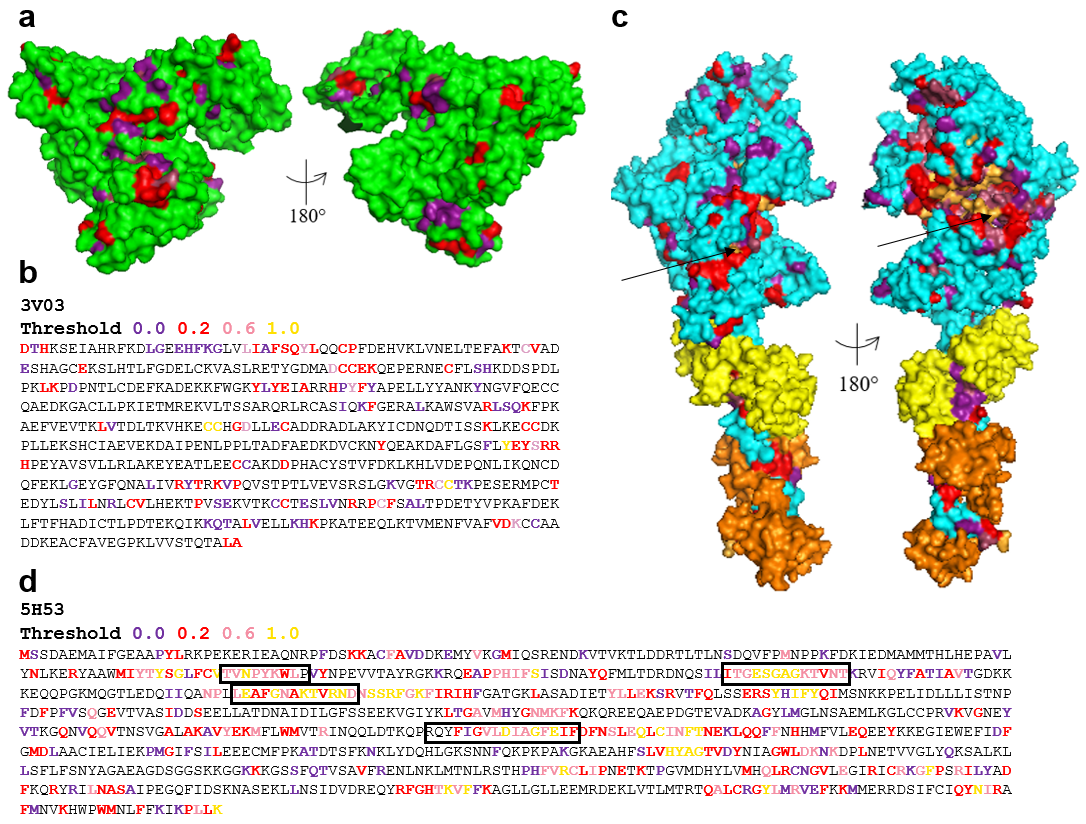


**Fig. S16. Surface availability on BSA and myosin II S1 for ATP binding as calculated using ATPint for different thresholds.** **a** Model of BSA with predicted cumulative surface availability for ATP binding as calculated using ATPint for different thresholds (**0.0 0.2 0.6 1.0)**. **b** Amino acid sequence of BSA with binding residues as recognized under different ATPint threshold values. Note: since it is known that BSA does interact with ATP there needs to be clearly defined interacting surfaces for that. Only by using ATPint with a threshold of 0.2 we could detect such surfaces. The other higher thresholds are too high and only show few scattered residues. **c** Model of S1 with predicted cumulative surface availability for ATP binding as calculated using ATPint for different thresholds (**0.0 0.2 0.6 1.0)** suggesting a “highway” for ATP transport to the active site. **d** Amino acid sequence of S1 with binding residues as recognized under different threshold values. Black squares represent residues of active ATP binding site. Note that increasing the threshold makes the highway slowly disappear. However the highway, though less evident, is still present with a threshold of 0.6 (see arrows in c). See further SI text section 2.9.

# 3. Supplementary References

1 Cordes, T., Vogelsang, J. & Tinnefeld, P. On the mechanism of Trolox as antiblinking and antibleaching reagent. *J Am Chem Soc* **131**, 5018-5019, doi:10.1021/ja809117z (2009).

2 Rahman, M. A., Usaj, M., Rassier, D. E. & Mansson, A. Blebbistatin Effects Expose Hidden Secrets in the Force-Generating Cycle of Actin and Myosin. *Biophys J* **115**, 386-397, doi:10.1016/j.bpj.2018.05.037 (2018).

3 Rahman, M. A., Salhotra, A. & Månsson, A. Comparative analysis of widely used methods to remove nonfunctional myosin heads for the in vitro motility assay. *Journal of muscle research and cell motility* **39**, 175-187, doi:10.1007/s10974-019-09505-1 (2018).

4 Swoboda, M. *et al.* Enzymatic oxygen scavenging for photostability without pH drop in single-molecule experiments. *ACS Nano* **6**, 6364-6369, doi:10.1021/nn301895c (2012).

5 Waterman-Storer, C. M. Microtubule/Organelle Motility Assays. *Current Protocols in Cell Biology* **00**, 13.11.11-13.11.21, doi:10.1002/0471143030.cb1301s00 (1998).

6 Na3VO4 Stock Solution. *Cold Spring Harbor Protocols* **2017**, pdb.rec092940, doi:10.1101/pdb.rec092940 (2017).

7 Martin-Fernandez, M. L., Tynan, C. J. & Webb, S. E. A 'pocket guide' to total internal reflection fluorescence. *J Microsc* **252**, 16-22, doi:10.1111/jmi.12070 (2013).

8 Kwakwa, K. *et al.* easySTORM: a robust, lower-cost approach to localisation and TIRF microscopy. *J Biophotonics* **9**, 948-957, doi:10.1002/jbio.201500324 (2016).

9 Fischer, A. H., Jacobson, K. A., Rose, J. & Zeller, R. Preparation of slides and coverslips for microscopy. *CSH Protoc* **2008**, pdb prot4988, doi:10.1101/pdb.prot4988 (2008).

10 Sundberg, M. *et al.* Silanized surfaces for in vitro studies of actomyosin function and nanotechnology applications. *Anal Biochem* **323**, 127-138, doi:10.1016/j.ab.2003.07.022 (2003).

11 Albet-Torres, N. *et al.* Mode of heavy meromyosin adsorption and motor function correlated with surface hydrophobicity and charge. *Langmuir* **23**, 11147-11156, doi:10.1021/la7008682 (2007).

12 Benoit, M. & Sosa, H. Use of Single Molecule Fluorescence Polarization Microscopy to Study Protein Conformation and Dynamics of Kinesin-Microtubule Complexes. *Methods Mol Biol* **1665**, 199-216, doi:10.1007/978-1-4939-7271-5_11 (2018).

13 Persson, M. *et al.* Transportation of nanoscale cargoes by myosin propelled actin filaments. *PLoS One* **8**, e55931, doi:10.1371/journal.pone.0055931 (2013).

14 Månsson, A. & Tagerud, S. Multivariate statistics in analysis of data from the in vitro motility assay. *Anal Biochem* **314**, 281-293, doi:10.1016/s0003-2697(02)00610-3 (2003).

15 Balaz, M., Sundberg, M., Persson, M., Kvassman, J. & Månsson, A. Effects of Surface Adsorption on Catalytic Activity of Heavy Meromyosin Studied using Fluorescent ATP Analogue. *Biochemistry* **46**, 7233-7251 (2007).

16 Persson, M. *et al.* Heavy Meromyosin Molecules Extend more than 50 nm above Adsorbing Electronegative Surfaces. *Langmuir* **26**, 9927-9936 (2010).

17 Verbrugge, S., Lechner, B., Woehlke, G. & Peterman, E. J. Alternating-site mechanism of kinesin-1 characterized by single-molecule FRET using fluorescent ATP analogues. *Biophys J* **97**, 173-182, doi:10.1016/j.bpj.2009.02.073 (2009).

18 Amrute-Nayak, M., Antognozzi, M., Scholz, T., Kojima, H. & Brenner, B. Inorganic phosphate binds to the empty nucleotide binding pocket of conventional myosin II. *J Biol Chem* **283**, 3773-3781, doi:10.1074/jbc.M706779200 (2008).

19 Amrute-Nayak, M. *et al.* ATP turnover by individual myosin molecules hints at two conformers of the myosin active site. *Proceedings of the National Academy of Sciences of the United States of America* **111**, 2536-2541, doi:10.1073/pnas.1316390111 (2014).

20 Bain, F. E., Wu, C. G. & Spies, M. Single-molecule sorting of DNA helicases. *Methods* **108**, 14-23, doi:10.1016/j.ymeth.2016.05.009 (2016).

21 Boehm, E. M., Subramanyam, S., Ghoneim, M., Washington, M. T. & Spies, M. Quantifying the Assembly of Multicomponent Molecular Machines by Single-Molecule Total Internal Reflection Fluorescence Microscopy. *Methods Enzymol* **581**, 105-145, doi:10.1016/bs.mie.2016.08.019 (2016).

22 Schindelin, J. *et al.* Fiji: an open-source platform for biological-image analysis. *Nat Methods* **9**, 676-682, doi:10.1038/nmeth.2019 (2012).

23 Rueden, C. T. *et al.* ImageJ2: ImageJ for the next generation of scientific image data. *BMC Bioinformatics* **18**, 529, doi:10.1186/s12859-017-1934-z (2017).

24 Abramoff, M., Magalhães, P. & Ram, S. J. Image Processing with ImageJ. *Biophotonics International* **11**, 36-42 (2003).

25 Schneider, C. A., Rasband, W. S. & Eliceiri, K. W. NIH Image to ImageJ: 25 years of image analysis. *Nat Methods* **9**, 671-675, doi:10.1038/nmeth.2089 (2012).

26 Zananiri, R. *et al.* Auxiliary ATP binding sites power rapid unwinding by RecBCD. *bioRxiv*, 210823, doi:10.1101/210823 (2018).

27 Chauhan, J. S., Mishra, N. K. & Raghava, G. P. Identification of ATP binding residues of a protein from its primary sequence. *BMC Bioinformatics* **10**, 434, doi:10.1186/1471-2105-10-434 (2009).

28 Schrodinger, LLC. *The PyMOL Molecular Graphics System, Version 1.8* (2015).

29 Hu, J., Li, Y., Zhang, Y. & Yu, D. J. ATPbind: Accurate Protein-ATP Binding Site Prediction by Combining Sequence-Profiling and Structure-Based Comparisons. *J Chem Inf Model* **58**, 501-510, doi:10.1021/acs.jcim.7b00397 (2018).

30 Yu, D. J. *et al.* TargetATPsite: a template-free method for ATP-binding sites prediction with residue evolution image sparse representation and classifier ensemble. *J Comput Chem* **34**, 974-985, doi:10.1002/jcc.23219 (2013).

31 Thangudu, R. R. *et al.* Knowledge-based annotation of small molecule binding sites in proteins. *BMC Bioinformatics* **11**, 365, doi:10.1186/1471-2105-11-365 (2010).

32 Bauer, M., Baumann, J. & Trommer, W. E. ATP binding to bovine serum albumin. *FEBS Lett* **313**, 288-290, doi:10.1016/0014-5793(92)81211-4 (1992).

33 Takeda, S., Miyauchi, S., Nakayama, H. & Kamo, N. Adenosine 5'-triphosphate binding to bovine serum albumin. *Biophys Chem* **69**, 175-183, doi:10.1016/s0301-4622(97)00084-7 (1997).

34 Vandenberk, N., Barth, A., Borrenberghs, D., Hofkens, J. & Hendrix, J. Evaluation of Blue and Far-Red Dye Pairs in Single-Molecule Förster Resonance Energy Transfer Experiments. *The Journal of Physical Chemistry B* **122**, 4249-4266, doi:10.1021/acs.jpcb.8b00108 (2018).

35 Bartley, L. E., Zhuang, X., Das, R., Chu, S. & Herschlag, D. Exploration of the Transition State for Tertiary Structure Formation between an RNA Helix and a Large Structured RNA. *Journal of Molecular Biology* **328**, 1011-1026, doi:https://doi.org/10.1016/S0022-2836(03)00272-9 (2003).

36 Karlsson, J. K. G., Laude, A., Hall, M. J. & Harriman, A. Photo-isomerization of the Cyanine Dye Alexa-Fluor 647 (AF-647) in the Context of dSTORM Super-Resolution Microscopy. *Chemistry – A European Journal* **25**, 14983-14998, doi:10.1002/chem.201904117 (2019).

37 Dave, R., Terry, D. S., Munro, J. B. & Blanchard, S. C. Mitigating unwanted photophysical processes for improved single-molecule fluorescence imaging. *Biophys J* **96**, 2371-2381, doi:10.1016/j.bpj.2008.11.061 (2009).

38 Gust, A. *et al.* A starting point for fluorescence-based single-molecule measurements in biomolecular research. *Molecules* **19**, 15824-15865, doi:10.3390/molecules191015824 (2014).

39 Cordes, T., Maiser, A., Steinhauer, C., Schermelleh, L. & Tinnefeld, P. Mechanisms and advancement of antifading agents for fluorescence microscopy and single-molecule spectroscopy. *Phys Chem Chem Phys* **13**, 6699-6709, doi:10.1039/c0cp01919d (2011).

40 Goodno, C. C. & Taylor, E. W. Inhibition of actomyosin ATPase by vanadate. *Proceedings of the National Academy of Sciences of the United States of America* **79**, 21-25, doi:10.1073/pnas.79.1.21 (1982).

41 Caremani, M., Lehman, S., Lombardi, V. & Linari, M. Orthovanadate and orthophosphate inhibit muscle force via two different pathways of the myosin ATPase cycle. *Biophys J* **100**, 665-674, doi:10.1016/j.bpj.2010.12.3723 (2011).

42 Zhang, Z., Yang, Y., Tang, X., Chen, Y. & You, Y. Effects of Ionic Strength on Chemical Forces and Functional Properties of Heat-induced Myofibrillar Protein Gel. *Food Science and Technology Research* **21**, 597-605, doi:10.3136/fstr.21.597 (2015).

43 Hu, Y. *et al.* Dye adsorption by resins: Effect of ionic strength on hydrophobic and electrostatic interactions. *Chemical Engineering Journal* **228**, 392-397, doi:https://doi.org/10.1016/j.cej.2013.04.116 (2013).

44 Shoemaker, B. A. *et al.* Inferred Biomolecular Interaction Server--a web server to analyze and predict protein interacting partners and binding sites. *Nucleic Acids Res* **38**, D518-524, doi:10.1093/nar/gkp842 (2010).

45 Shoemaker, B. A. *et al.* IBIS (Inferred Biomolecular Interaction Server) reports, predicts and integrates multiple types of conserved interactions for proteins. *Nucleic Acids Res* **40**, D834-840, doi:10.1093/nar/gkr997 (2012).

46 Kozakov, D. *et al.* The FTMap family of web servers for determining and characterizing ligand-binding hot spots of proteins. *Nat Protoc* **10**, 733-755, doi:10.1038/nprot.2015.043 (2015).

47 Chen, K., Mizianty, M. J. & Kurgan, L. Prediction and analysis of nucleotide-binding residues using sequence and sequence-derived structural descriptors. *Bioinformatics* **28**, 331-341, doi:10.1093/bioinformatics/btr657 (2012).
