# Supplementary material for: Single molecule turnover of fluorescent ATP by myosin and actomyosin unveil elusive enzymatic mechanisms
Source: Commun Biol. 2021 Jan 13;4:64. doi: 10.1038/s42003-020-01574-0 (PMC7806905; doi:10.1038/s42003-020-01574-0)
Supplement: Supplementary file 3 — Description of Additional Supplementary Files [file 42003_2020_1574_MOESM3_ESM.pdf]

Description of Additional Supplementary Files

File name: Supplementary Movie S1

Description: Myosin basal ATPase. Time laps TIRF microscopy image sequence of Alexa647-ATP binding to HMM deposited via actin filament. The ~15 min video corresponds to the data presented in Fig. 2b, ii. The dashed line indicates the original position of the actin filament mediating the HMM deposition. The video was accelerated ~5 times (from 19 fps to 100 fps for practical reasons. Bar represents 5 µm.

File name: Supplementary Movie S2

Description: Actomyosin ATPase. Time laps TIRF microscopy image sequence of Alexa647-ATP binding to actomyosin. HMM was deposited via actin filament. The ~15min video corresponds to the data presented in Fig. 2b, iii. The dashed lines indicate the position of the actin filaments. The video was accelerated ~5 times (from 19 fps to 100 fps for better presentation. Bar represents 5 µm.

File name: Supplementary Data Figure 2

Description: The data to reproduce graphs under Figure 2

File name: Supplementary Data Figure 3

Description: The data to reproduce graphs under Figure 3

File name: Supplementary Data Figure 4

Description: The data to reproduce graphs under Figure 4

File name: Supplementary Data Figure 5

Description: The data to reproduce graphs under Figure 5

File name: Supplementary Data Figure 6

Description: The data to reproduce graphs under Figure 6
