# Supplementary material for: Single molecule turnover of fluorescent ATP by myosin and actomyosin unveil elusive enzymatic mechanisms
Source: Commun Biol. 2021 Jan 13;4:64. doi: 10.1038/s42003-020-01574-0 (PMC7806905; doi:10.1038/s42003-020-01574-0)
Supplement: Supplementary file 11 — Reporting Summary [file 42003_2020_1574_MOESM11_ESM.pdf]

nature research

Corresponding author(s): Alf Månsson, Marko Ušaj

Last updated by author(s): Nov 3, 2020

Reporting Summary

Nature Research wishes to improve the reproducibility of the work that we publish. This form provides structure for consistency and transparency in reporting. For further information on Nature Research policies, see Authors & Referees and the Editorial Policy Checklist.

Statistics

For all statistical analyses, confirm that the following items are present in the figure legend, table legend, main text, or Methods section.

n/a	Confirmed	
☐	☒	The exact sample size (n) for each experimental group/condition, given as a discrete number and unit of measurement
☒	☐	A statement on whether measurements were taken from distinct samples or whether the same sample was measured repeatedly
☒	☐	The statistical test(s) used AND whether they are one- or two-sided<br>*Only common tests should be described solely by name; describe more complex techniques in the Methods section.*
☒	☐	A description of all covariates tested
☐	☒	A description of any assumptions or corrections, such as tests of normality and adjustment for multiple comparisons
☐	☒	A full description of the statistical parameters including central tendency (e.g. means) or other basic estimates (e.g. regression coefficient) AND variation (e.g. standard deviation) or associated estimates of uncertainty (e.g. confidence intervals)
☒	☐	For null hypothesis testing, the test statistic (e.g. F, t, r) with confidence intervals, effect sizes, degrees of freedom and P value noted<br>*Give P values as exact values whenever suitable.*
☒	☐	For Bayesian analysis, information on the choice of priors and Markov chain Monte Carlo settings
☒	☐	For hierarchical and complex designs, identification of the appropriate level for tests and full reporting of outcomes
☒	☐	Estimates of effect sizes (e.g. Cohen's d, Pearson's r), indicating how they were calculated

Our web collection on statistics for biologists contains articles on many of the points above.

Software and code

Policy information about availability of computer code

Data collection	NIS Elements (Nikon, ver. 4.51), HCImage (Hamamatsu Corporation, ver. 4.3.1.33)
Data analysis	Matlab with toolboxes and scripts within (ver. 2017, 2018a, 2019b), Fiji (up to ver. 1.52s), MS Excel 2016, Graphpad Prism (ver. 7 and 8), PyMOL molecular graphic system (ver. 1.2r3pre), ATPint (online server), PDB (online server).

For manuscripts utilizing custom algorithms or software that are central to the research but not yet described in published literature, software must be made available to editors/reviewers. We strongly encourage code deposition in a community repository (e.g. GitHub). See the Nature Research guidelines for submitting code & software for further information.

Data

Policy information about availability of data

All manuscripts must include a data availability statement. This statement should provide the following information, where applicable:
- Accession codes, unique identifiers, or web links for publicly available datasets
- A list of figures that have associated raw data
- A description of any restrictions on data availability

The data that support the findings of this study are available from the corresponding authors upon reasonable request.

Field-specific reporting

Please select the one below that is the best fit for your research. If you are not sure, read the appropriate sections before making your selection.

☒ Life sciences ☐ Behavioural & social sciences ☐ Ecological, evolutionary & environmental sciences

Life sciences study design

All studies must disclose on these points even when the disclosure is negative.

Sample size	No sample size calculation was performed prior to the experiments as the appropriate sample size for this type of experiments has been determined previously (Amrute-Nayak et al., PNAS, 2014, 111:2536-41, Rahman et al., J Muscle Research Cell Motility, 2018, 39:175–187).
Data exclusions	Data were only excluded based on the strictly applied criteria that each individual trace representing one myosin molecule must contain at least 10 independent events per 15 min trace. This criterion was adopted from a previous study (Amrute-Nayak et. al. PNAS, 2014, 111:2536-41) to standardize our work to recent approaches.
Replication	We are not quite sure that the issue of replication is applicable to the analyses in the current study as we construct distributions of lots of events as basis for further quantitative analysis. Nevertheless, with the aim to clarify this point we try to explain how the analysis was performed. Generally, based on previous work, each binding event of a fluorescent ATP molecule to a myosin molecule was assumed to be an independent random event independent of the experimental occasion, or myosin batch. The duration of these events were then used to produce probability distributions that were analyzed to give the quantitative data based on exponential fits to the distributions. The exponential model has strong theoretical foundations in this regard. The number of events used for construction of the distributions ranged between about 100 and 1000 with limited effects of the sample size on the quantitative characteristics of the distributions. This finding supports our assumption that all individual events are independent. Support for that assumption also derives from previous findings of very similar kinetic properties of myosin from different preparations (Persson et al., Biophysical Journal, 2013, 105:1871-81; Rahman et al., J Muscle Research Cell Motility, 2018, 39:175–187). Furthermore, the assumption is supported by similar results (within the range that support our conclusions) in experiments performed on different days using different myosin preparations (Fig. S6 and comparison of Fig. 1h,i and Fig. S8). Finally, in support of our assumed definition of independent events, no consistent changes in behavior were seen during the course of a given experiment (Fig. S9a-b).
Randomization	Our study does not include sample allocations in different groups. When we were collecting microscopy data from sample (imaging chamber), fields of view were chosen randomly. After such choice all events were analyzed provided that they met predefined criteria (see above under data exclusions)
Blinding	The experiments were performed by skilled persons. Blinding is not relevant in our study due to nature of experiments, extra time and cost which would need to train additional PhD students, postdocs and researchers. Thus most of the experiments were performed by a single person which collect and analyzed the data. However, parts of the in vitro motility assays and single molecule experiments were collected by one and analyzed by another person. Some data were also analyzed in duplicate by different persons with negligible differences in results.

Reporting for specific materials, systems and methods

We require information from authors about some types of materials, experimental systems and methods used in many studies. Here, indicate whether each material, system or method listed is relevant to your study. If you are not sure if a list item applies to your research, read the appropriate section before selecting a response.

Materials & experimental systems

n/a	Involved in the study
☒	☐ Antibodies
☒	☐ Eukaryotic cell lines
☒	☐ Palaeontology
☐	☒ Animals and other organisms
☒	☐ Human research participants
☒	☐ Clinical data

Methods

n/a	Involved in the study
☒	☐ ChIP-seq
☒	☐ Flow cytometry
☒	☐ MRI-based neuroimaging

Animals and other organisms

Policy information about studies involving animals; ARRIVE guidelines recommended for reporting animal research

Laboratory animals	New Zealand white rabbits (female, 2 kg, 8-9 months).
Wild animals	The study did not involve wild animals.
Field-collected samples	The study did not involve field-collected samples.
Ethics oversight	Regional Ethical Committee for Animal experiments in Linköping, Sweden, reference number 73-14.

Note that full information on the approval of the study protocol must also be provided in the manuscript.
